# A rule-based method for predicting the electrical activation of the heart with cardiac resynchronization therapy from non-invasive clinical data

**DOI:** 10.1016/j.media.2019.06.017

**Published:** 2019-07-05

**Authors:** A.W.C. Lee, U.C. Nguyen, O. Razeghi, J. Gould, B.S. Sidhu, B. Sieniewicz, J. Behar, M. Mafi-Rad, G. Plank, F.W. Prinzen, C.A. Rinaldi, K. Vernooy, S. Niederer

**Affiliations:** a School of Biomedical Engineering and Imaging Sciences, King’s College London, London, United Kingdom; b Department of Physiology, Maastricht University Medical Center (MUMC+), Cardiovascular Research Institute Maastricht (CARIM), Maastricht, the Netherlands; c Department of Cardiology, Maastricht University Medical Center (MUMC+), Cardiovascular Research Institute Maastricht (CARIM), Maastricht, the Netherlands; d Bart’s Heart Centre, St. Bartholomew’s Hospital, London, United Kingdom; e Department of Biophysics, Medical University of Graz, Graz, Austria; f Department of Cardiology, Radboud University Medical Center, Nijmegen, the Netherlands

**Keywords:** Cardiac resynchronization therapy, Electrophysiology, Computational models, Patient-specific simulations

## Abstract

**Background:**

Cardiac Resynchronization Therapy (CRT) is one of the few effective treatments for heart failure patients with ventricular dyssynchrony. The pacing location of the left ventricle is indicated as a determinant of CRT outcome.

**Objective:**

Patient specific computational models allow the activation pattern following CRT implant to be predicted and this may be used to optimize CRT lead placement.

**Methods:**

In this study, the effects of heterogeneous cardiac substrate (scar, fast endocardial conduction, slow septal conduction, functional block) on accurately predicting the electrical activation of the LV epicardium were tested to determine the minimal detail required to create a rule based model of cardiac electrophysiology. Non-invasive clinical data (CT or CMR images and 12 lead ECG) from eighteen patients from two centers were used to investigate the models.

**Results:**

Validation with invasive electro-anatomical mapping data identified that computer models with fast endocardial conduction were able to predict the electrical activation with a mean distance errors of 9.2 ± 0.5 mm (CMR data) or (CT data) 7.5 ± 0.7 mm.

**Conclusion:**

This study identified a simple rule-based fast endocardial conduction model, built using non-invasive clinical data that can be used to rapidly and robustly predict the electrical activation of the heart. Pre-procedural prediction of the latest electrically activating region to identify the optimal LV pacing site could potentially be a useful clinical planning tool for CRT procedures.

## 1 Introduction

Cardiac resynchronization therapy (CRT) has emerged as an effective therapy for heart failure patients with ventricular conduction disturbances, such as left bundle branch block (LBBB), resulting in a dyssynchronous ventricular activation. CRT aims to restore this dyssynchronous activation and the location of the left ventricular (LV) pacing lead has repeatedly been identified as an important contributor to patient response (Singh et al., 2011; Ypenburg et al., 2008).

The optimal pacing site has been proposed to be located at the latest activating regions (Khan et al., 2012; Saba et al., 2013; Zanon et al., 2014) and outside of scar (Khan et al., 2009; Leyva et al., 2011; Singh et al., 2011). However, this approach has two short comings. Firstly, in cases where scar tissue causes a circumferential block in the activation wave, the latest region to activate can be located adjacent to scar. Secondly, the optimal location is based on the state of the patient prior to implant and is determined without consideration of the location of the right ventricle (RV) pacing lead, which may alter the location of the latest activating region (Mafi Rad et al., 2014). Recent studies have found that increased rate of endocardial activation patterns (Sohal et al., 2015) and reduced QRS duration (Derval et al., 2014) result in an improved response to CRT, this could potentially provide a better cost function for optimizing optimal pacing location.

As the heart is paced from the RV and LV electrodes that can be positioned independently, arguably pacing locations need to be optimized not on the intrinsic activation pattern in the heart prior to CRT, but taking into account the effect of both leads on the activation pattern once CRT is implanted. Optimizing therapy planning using the activation pattern post implant requires the ability to predict how different pacing configurations will alter the activation pattern for given lead locations.

Computer models of cardiac electrophysiology have been developed to perform patient specific simulations to predict electrical activation patterns. This approach has been used for predicting changes in activation patterns in CRT and to investigate the optimal atrioventricular delay, interventricular delay and lead locations (Lee et al., 2017; Miri et al., 2009; Reumann et al., 2007a, 2007b; Villongco et al., 2016).

The muscle fiber directions within the heart are important in determining the pattern of electrical activation throughout the ventricles, as the electrical activation spreads predominantly along the direction of the myofibers. Measurements of the fiber angles (Holmes et al., 2000; Scollan et al., 1998; Seemann et al., 2006) have found the fiber orientation to be relatively consistent in the ventricles and well described by rule-based methods (Bayer et al., 2012). Therefore, in recent years, studies have adopted rule-based fiber methods to approximate the fiber directions in electrophysiology models of human hearts (Aguado-Sierra et al., 2011; Crozier et al., 2015; Kayvanpour et al., 2015; Kerckhoffs et al., 2012; Lee et al., 2017; Okada et al., 2017; Sermesant et al., 2012; Tobon-Gomez et al., 2013; Villongco et al., 2016).

Previous models have personalized ventricular activation and predicted activation patterns for CRT using invasive measures of the electrical properties of the heart, such as electro-anatomical mapping (EAM) of the epicardium via the coronary veins or the endocardium by contact or non-contact mapping (Aguado-Sierra et al., 2011; Crozier et al., 2015; Lee et al., 2017; Sermesant et al., 2012; Tobon-Gomez et al., 2013; Villongco et al., 2016). These studies required invasive data, collected intra-procedurally, meaning that these models are unable to be used for prospective prediction of the electrical activation of the heart or to track the response to CRT.

In recent studies, non-invasive ECG data from conventional 12 lead ECGs or from body surface potential mapping (with commercial versions recording up to 256 ECGs) were used to parameterize electrophysiology models (Giffard-Roisin et al., 2017; Kayvanpour et al., 2015; Okada et al., 2017). In these studies, the cardiac substrate was assumed to be homogeneous with uniform conduction velocity (Giffard-Roisin et al., 2017) or with fast endocardial conductivity (Kayvanpour et al., 2015; Okada et al., 2017). These studies used non-invasive data to constrain the models, however these modeling frameworks required complex and iterative fitting to parameterize the onset site/s of the electrical activation (Giffard-Roisin et al., 2017; Okada et al., 2017) and the conductivities of the myocardium (Giffard-Roisin et al., 2017) and the fast conducting endocardium (Kayvanpour et al., 2015; Okada et al., 2017).

A rule-based method for defining the electrophysiology parameters would be useful in facilitating the development of electrophysiology models in individual patients. However, the material properties of the heart have been less well described, with experimental studies reporting a range of conduction velocities (0.07–0.75 m/s) along the fiber direction, with a 2–7 fold increase in electrical conduction velocity along longitudinal direction in comparison to the transverse direction of the myofibers (Clerc, 1976; Roberts et al., 1979; Roth, 1997).

Experimental studies have also found heterogeneous conduction velocities throughout the ventricles. In addition to the three to six fold increase in the conduction velocity in the Purkinje system (Draper and Mya-Tu, 1959; Myerburg et al., 1978), the sub-endocardial layer has also been found to have a 1.5–4 fold increased electrical conduction velocity in comparison to the normal myocardium (Draper and Mya-Tu, 1959; Myerburg et al., 1978; Strik et al., 2011). In experimental studies of the canine heart, the spread of the electrical activation in the endocardium was found to start simultaneously across the RV and starting in the bottom third of the LV and spreading in an apical to basal direction in the LV (Myerburg et al., 1972), while in human hearts activation in the LV began from endocardial areas in the lower third of the free wall, mid septal wall and in the basal area underneath the mitral valve (Durrer et al., 1970). A number of electrophysiology simulation studies have thus incorporated a fast endocardial layer representing the Purkinje network or the increased conduction velocity in the sub-endocardial layer (Aguado-Sierra et al., 2011; Hyde et al., 2015; Kayvanpour et al., 2015; Okada et al., 2017).

Additional heterogeneities have been reported to influence activation. The conduction velocity of scarred myocardium have been found to be reduced in comparison to normal tissue (de Bakker et al., 1993; Peters et al., 1993). Clinical studies have also found that the electrical activation pattern in the LV can be classed as type I/II in patients suffering from LBBB, with non-contact mapping studies showing a C-shaped or U-shaped pattern of electrical activation where there is a region of functional block in the anterior or posterior regions of the LV (Auricchio et al., 2004; Jia et al., 2006). A decrease in the conduction velocity has also been observed in the septum in canines and humans with LBBB (Prinzen and Auricchio, 2008; Sohal et al., 2014; Strik et al., 2013). These heterogeneities have been reported across different pathologies and species, however their relative importance in predicting the electrical activation pattern has not been determined.

In this study we investigated the impact of scar, slow septal conduction, fast endocardial conduction and the presence of type I/II activation patterns on predicting cardiac electrophysiology for CRT patients using simple models of tissue heterogeneity. The simplest rule-based methods for defining the electrical heterogeneities present in the ventricles needed to robustly predict the activation patterns based on non-invasive imaging of the patient anatomy and ECG measurements was identified.

## 2 Methods

### 2.1 Study population

This study was conducted on data collected from fourteen patients who underwent a *de novo* CRT device implantation at the Maastricht University Medical Center and underwent a cardiac magnetic resonance (CMR) imaging before implantation. An additional four RV-pacing patients upgrading to CRT were recruited at Guys and St Thomas’ Hospital. All patients had a I or II CRT indication according to the ESC guidelines (LV ejection fractions of < 35% with mild to severe heart failure symptoms with LBBB according to specific criteria or non-LBBB with a QRS duration of > 150 ms). Data was collected from patients at the two centers with the study protocols approved by the respective local ethics committees.

### 2.2 Non-invasive data

In the CMR data set, images were acquired with a 1.5–3T Philips scanner (Achieva/Ingenia/Intera) prior to device implantation. Steady state free precession CMR sequence (slice thickness 6–8 mm, field of view 320–384 mm, matrix 256–560 × 256–560) was used to image the heart in 2-chamber, 3-chamber, 4-chamber views as well as a short axis stack covering the entire LV. Contrast-enhanced (CE) CMR images were also acquired with a 2D gradient echo inversion recovery sequence (slice thickness 7–10 mm, field of view 300–384 mm, matrix 240–576 × 240–576). The CMR images were manually segmented using customized software (CAAS MRV3.4, Pie Medical Imaging) to personalize the cardiac anatomy. Areas of cardiac infarct were semi-automatically segmented from the CE CMR images with the full-width half maximum method (Nguyen et al., 2017).

In the four CT cases, 3D whole heart CT images (slice thickness 0.5 mm, field of view 185–280 mm x 185–280 mm x 118–191 mm, and in-plane resolution 0.36–0.54 mm) were acquired with a Philips iCT256 or Siemens Somatom Force scanner. RV paced patients were scanned and subsequently underwent a CRT upgrade procedure, where a LV lead is implanted into the coronary sinus (CS) for epicardial pacing. The Philips model-based automatic segmentation tool (Peters et al., 2007) was used to segment the ventricles and the main CS branch. Semi-automatic segmentation of the coronary branches was then performed using CRT segmentation software (Mountney et al., 2017).

Non-invasive measures of the electrical activation of the heart were obtained with 12 lead ECG during RV pacing. The QRS onset and duration from one of the frontal leads were used to estimate the start of the electrical activation and the total time of activation in the ventricles.

### 2.3 Electroanatomical mapping

At both centers, 3D EAM of the coronary veins was performed using Ensite NavX (St Jude Medical) intra-procedurally (Mafi Rad et al., 2014). In the *de novo* patient cases, an RV lead was implanted in the RV apex region, guided with fluoroscopy X-ray images and identified on the EAM. A unipolar sensing and pacing guidewire connected to the Ensite NaxX system was inserted into the CS and extended into the CS tributaries, to generate a 3D map of the CS and its tributaries as well as measuring the local activation times (LAT) of these sites with RV pacing. Across the 14 cases a large epicardial area of the LV free wall was mapped, as shown in Appendix A. The LAT, defined as the duration from onset of the QRS complex on the surface ECG to the steepest downslope on the intracardiac unipolar electrogram, was recorded throughout the procedure and was calculated as a percentage of the QRS duration. In the CMR data set, the EAM 3D anatomy was mapped to the segmentations of the CS using the fusion function of the Ensite NavX system as a post processing step by a clinician using the X-ray fluoroscopy images obtained during the procedure as guidance.

### 2.4 Electrical activation models

#### 2.4.1 CMR cases

Patient specific electrophysiology models were created from the 14 CMR data sets. The anatomy for each case was personalized from the segmentations. The location of the RV lead was identified from the EAM mapped onto the models using the fusion function in the Ensite NavX system. The locations where the LAT was measured with the guidewire were projected onto the closest point on the LV epicardial surface of the patient specific meshes with a root mean square error of 3.0–9.1 mm for the 14 CMR cases.

**Fig. 1:**
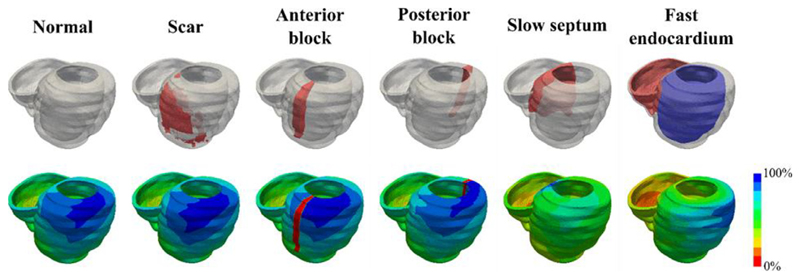
Six electrophysiology models were investigated with the inclusion of scar, functional block in the anterior or posterior regions of the LV, slow septal conduction, and fast endocardial conduction used to determine the importance of these factors in accurately simulating the electrical propagation across the ventricles.

A rule-based method was used to determine the fiber orientations (Bayer et al., 2012). The propagation of the electrical activation from pacing at the RV apex was simulated using an Reaction-Eikonal model (Neic et al., 2017) using the Cardiac Arrhythmia Research Package (CARP) (Niederer et al., 2011; Vigmond et al., 2003). The Eikonal conductivity term was fitted to match the QRS data using a grid search of low cost eikonal activation simulations. The Reaction-Eikonal conductivity was then input into the Reaction-Eikonal equations. This results in a predicted activation time error of no more than 1%. The conduction velocity was characterized by assuming the QRS duration was equal to the total activation time across the ventricles, with the computed QRS duration set to be within a tolerance of 5 ms. The conduction velocity in the ventricles was assumed to be transversely anisotropic, with the conduction velocity transverse to the fiber directions set to be 40% of the conduction velocity along the myofiber directions (Sermesant et al., 2012). The importance of including scar, slow septal conduction, fast endocardial conduction and the presence of type I/II activation patterns in predicting epicardial activation patterns were also explored using additional electrophysiology models [Fig. 1].

In the scar regions and functional block regions, the electrical activation was assumed to be completely blocked. While the slow septal conduction velocity was assumed to be 50% of the normal myocardium (Strik et al., 2013). A 1 mm thick fast endocardium layer was assumed to have a six-fold increased conduction velocity along the fiber direction compared to the normal myocardium (Draper and Mya-Tu, 1959; Myerburg et al., 1978).

#### 2.4.2 CT cases

Four patients were receiving an upgrade from RV pacing to CRT and were not eligible for CMR. CT whole heart images were segmented using Philips Model Based Segmentation Framework and used to create patient specific models. The location of the RV pacing lead was identified on the CT images. The coronary venous EAM was recorded intra-procedurally using a unipolar sensing and pacing guidewire connected to the Ensite NavX system. The anatomical 3D map was registered to the segmented coronary sinus from the CT images using CMISS (www.cmiss.org) host mesh fitting, with clinical guidance based on X-ray fluoroscopy imaging to determine which branches were mapped. As the segmentations of the CS and the patient specific meshes were derived from the same CT image, the mapped coronary venous EAM were also mapped onto the meshes. The locations where the electrical delay was measured with the guidewire were projected onto the closest point on the LV epicardial surface of the patient specific meshes with a root mean square error of 3.1–8.6 mm for the 4 CT cases.

The LAT from RV pacing was clinically measured with EAM in the coronary sinus tributaries. The models were evaluated by comparing the simulated LAT with intra-procedural clinical LAT measurements. The relative LAT error was taken as the difference between the model simulations and the clinical measurements. The temporal error for each simulation was calculated by multiplying the relative LAT error by the QRS duration for each case. Positive temporal error values indicate that the model activates slower than the clinical measurements, while negative values indicate that the model activates faster. The distance error measurement for each simulation was calculated as the minimum distance between the location of the EAM measurement projected onto the myocardium and the nearest tissue with an activation time that matches the measured activation time.

## 3 Results

**Fig. 2:**
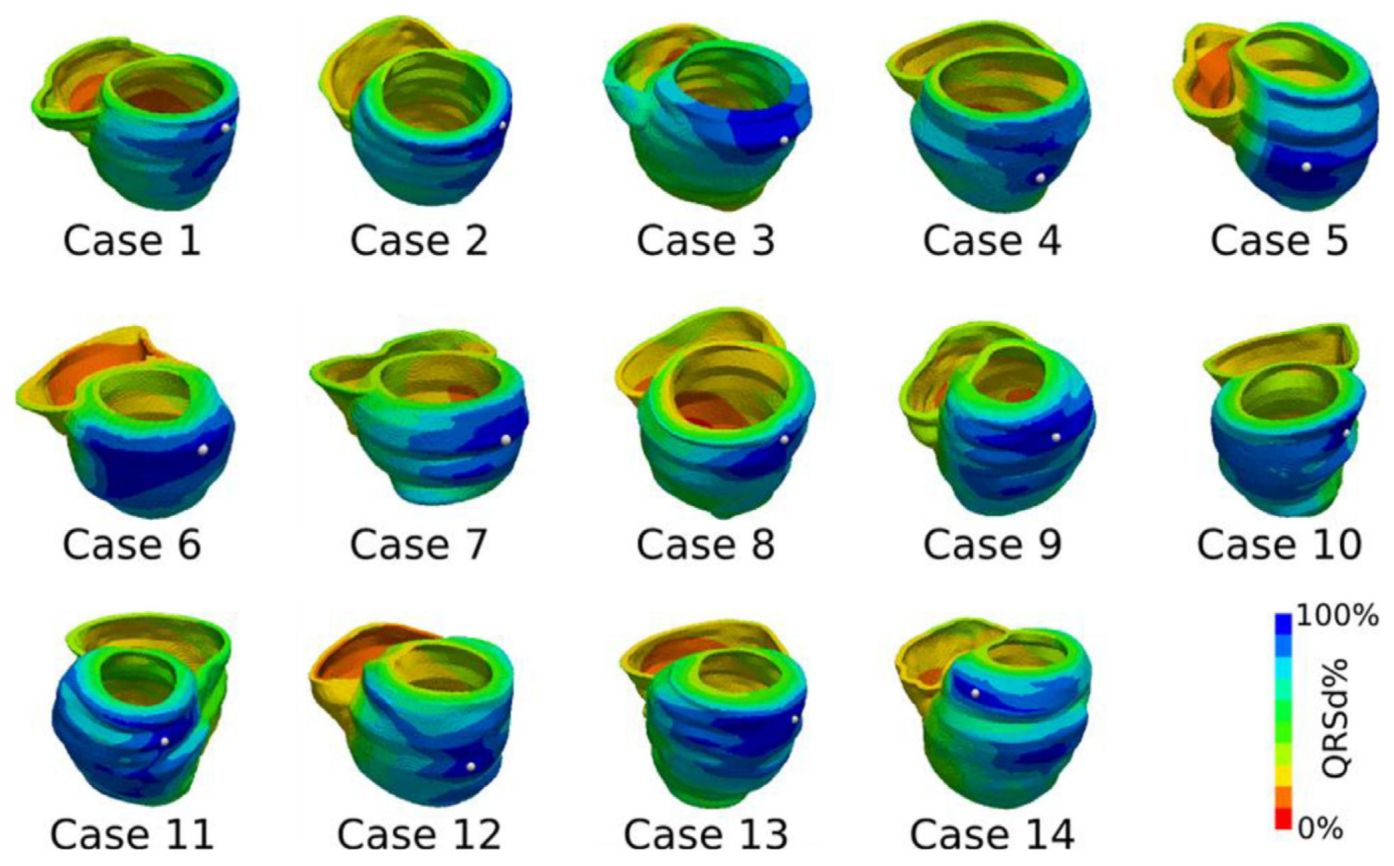
RV pacing with a six-fold increase in the ventricular endocardium was simulated for the 14CMR cases, with the electrical activation time normalized as a percentage of the QRS duration. The latest activated site is highlighted with a white dot.

**Fig. 3:**
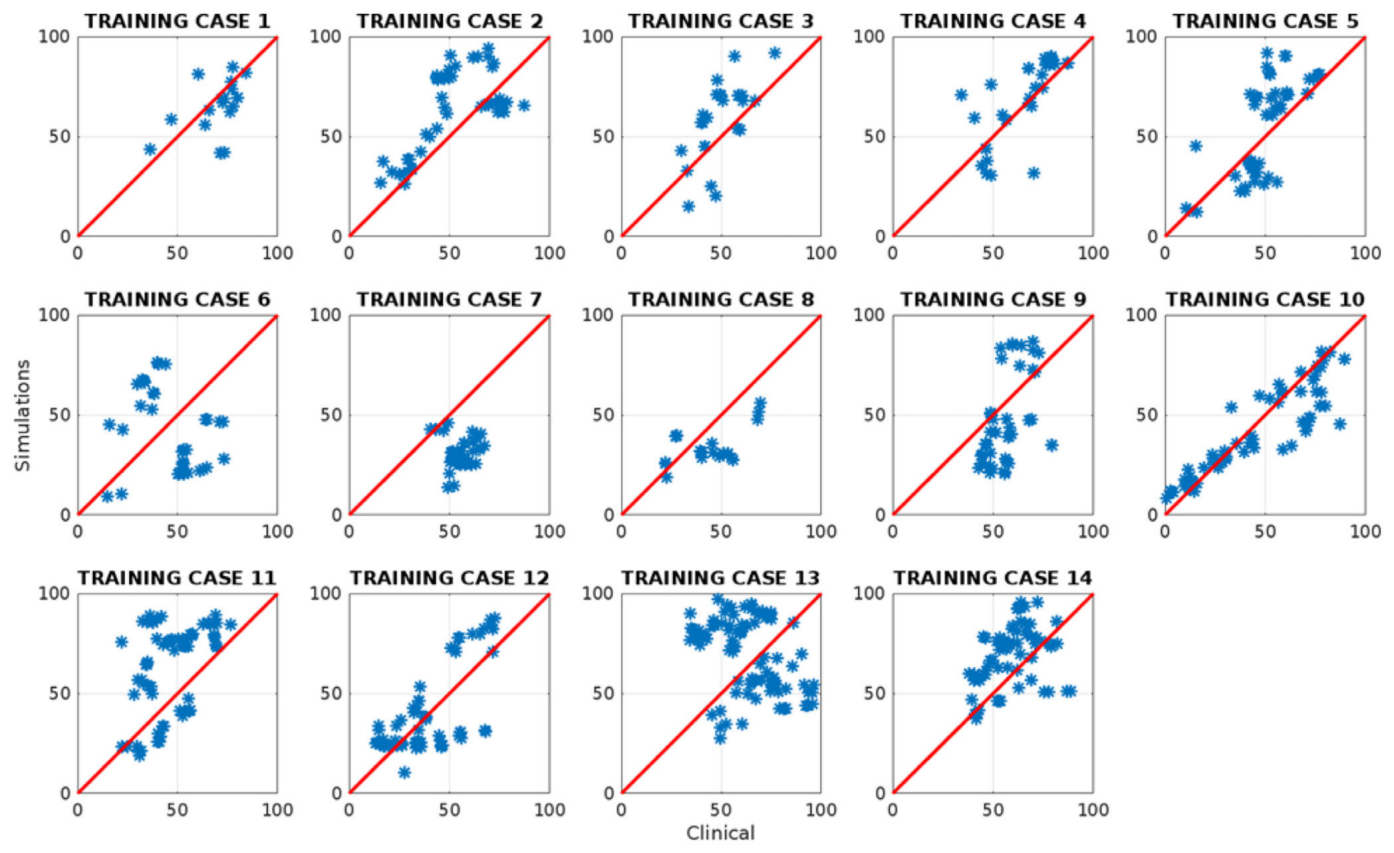
The simulated local activation time normalized as a percentage of the QRS duration (LAT) was compared against the clinical LAT for each of the measured sites in the coronary sinus venous branches for the model with fast endocardial conduction.

The electrical activation for each of the models was simulated for each of the 14 CMR cases with the conduction velocity along the fiber directions for the bulk myocardium characterized by the QRS duration. The propagation of the electrical activity from pacing at the RV for the six simple electrophysiology models (normal, with scar, with anterior or posterior block, with slow septal conduction and with fast endocardial conduction) were simulated as shown in Fig. 2. The LAT errors were computed for each model in each case as shown in Fig. 3.

**Fig. 4:**
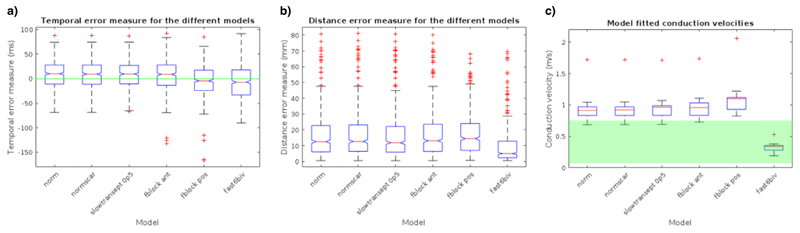
Simulations were run for the 14 CMR cases, with a basic model, inclusion of scar (for cases 9–14), slow septum, fast endocardial conduction, anterior or posterior functional block. Boxplots of the (a) temporal error, (b) distance error, and (c) conduction velocities are shown for each model. The conduction velocities which are between the physiologically plausible range (0.07–0.75 m/s) are highlighted in green.

The resulting temporal and distance errors for the six electrophysiology models are shown in Fig. 4. The results and analysis in the next section are from a leave-one-out cross validation, and the reported scores are the average of the mean temporal and distance errors. One-way ANOVA was used to compare the temporal and distance errors between the six simple electrophysiology models, and it was found that there were statistically significant differences in the temporal and distance errors for the models (p-value < 0.001). Tukey post-hoc tests indicated that only 2 models had significantly different temporal means (posterior functional block model (–5.1 ± 1.2 ms) and the fast endocardial conduction model (–7.0 ± 1.2 ms)), compared to the rest of the models (Normal: 7.6 ± 1.2 ms, Scar: 7.4 ± 1.2 ms, slow septum: 8.0 ± 1.2 ms, anterior functional block: 6.6 ± 1.2 ms), however the absolute temporal error mean values were comparable. Tukey post-hoc test indicated that the fast endocardial conduction model was the only model that had a significantly reduced mean distance error (9.2 mm ± 0.5 mm) in comparison to the other models (15.6–16.9 mm ± 0.5 mm) (see Appendix C).

To further constrain the model selection, several criteria were imposed on the solution of the models to ensure the simulations reflect known physiological constraints. In experimental studies, the conduction velocity of the ventricular myocardium has been found to range between 0.07–0.75 m/s (Clerc, 1976; Draper and Mya-Tu, 1959; Kleber and Rudy, 2004; Roberts et al., 1979; Sano et al., 1959; Weidmann, 1970), the models which extended outside this range were excluded from consideration. Endocardial mapping in LBBB cases have found the latest point of activation to lie close to the lateral wall of the LV (Auricchio et al., 2004), thus we also excluded models where the latest site of activation was in the septum. The comparison of model conduction velocity and physiological constraints is shown in Fig. 4C. The only model which fulfilled both criteria was the model with fast endocardial conduction. The mean temporal error for the models with six-fold fast endocardial conduction in the CMR cases was −7.0 ± 0.3 ms, mean distance error was 9.2 mm ± 0.1 mm, with mean model conduction velocity of 0.33 ± 0.08 m/s.

### 3.1 Sensitivity to model parameters

The sensitivity of the distance error measure to changes in the anatomy, fibre orientation, anisotropy ratio, and the slow septal conductivity were also investigated. It was found that regardless of changes in the anatomy, fibre orientation, anisotropy ratio, slow septal conductivity, the conclusion that fast endocardial conduction was the most important factor remained consistent (see Appendix B for details).

In addition to models with a six-fold increased conduction velocity in the endocardium, simulations were also run for up to ten-fold (Aguado-Sierra et al., 2011) increases in the endocardial conduction velocity (see Appendix B3). It was found that as the fast endocardial conduction ratio increased from 1.0 (normal model), the distance error between the model simulations and the clinical measurements gradually improved. We observed that there were no significant differences in the mean distance errors for five-fold to ten-fold increases in the FEC ratio (7.9–10.0 mm ± 0.4 mm). While the myocardial conduction velocity for all the fast endocardial conduction models remained within the values reported in experimental studies.

Experimental canine and human studies have shown that the normal depolarization of the heart proceeds in an apical to basal direction, with the greatest concentration of arborizations of the Purkinje network in the lower third of the heart in the apical-basal direction (Myerburg et al., 1972; Spach et al., 1963). Additional simulations were carried out with setting the fast endocardial conduction to be in the lower third of the heart. One-way ANOVA was used to compare the models with varying fast endocardial conduction ratios and it was found that, when the fast endocardial conduction was only located on the lower third of the heart, there were no differences in the mean distance error measures compared to the normal model (see Appendix B3).

## 4 Cross-modality validation

**Fig. 5:**
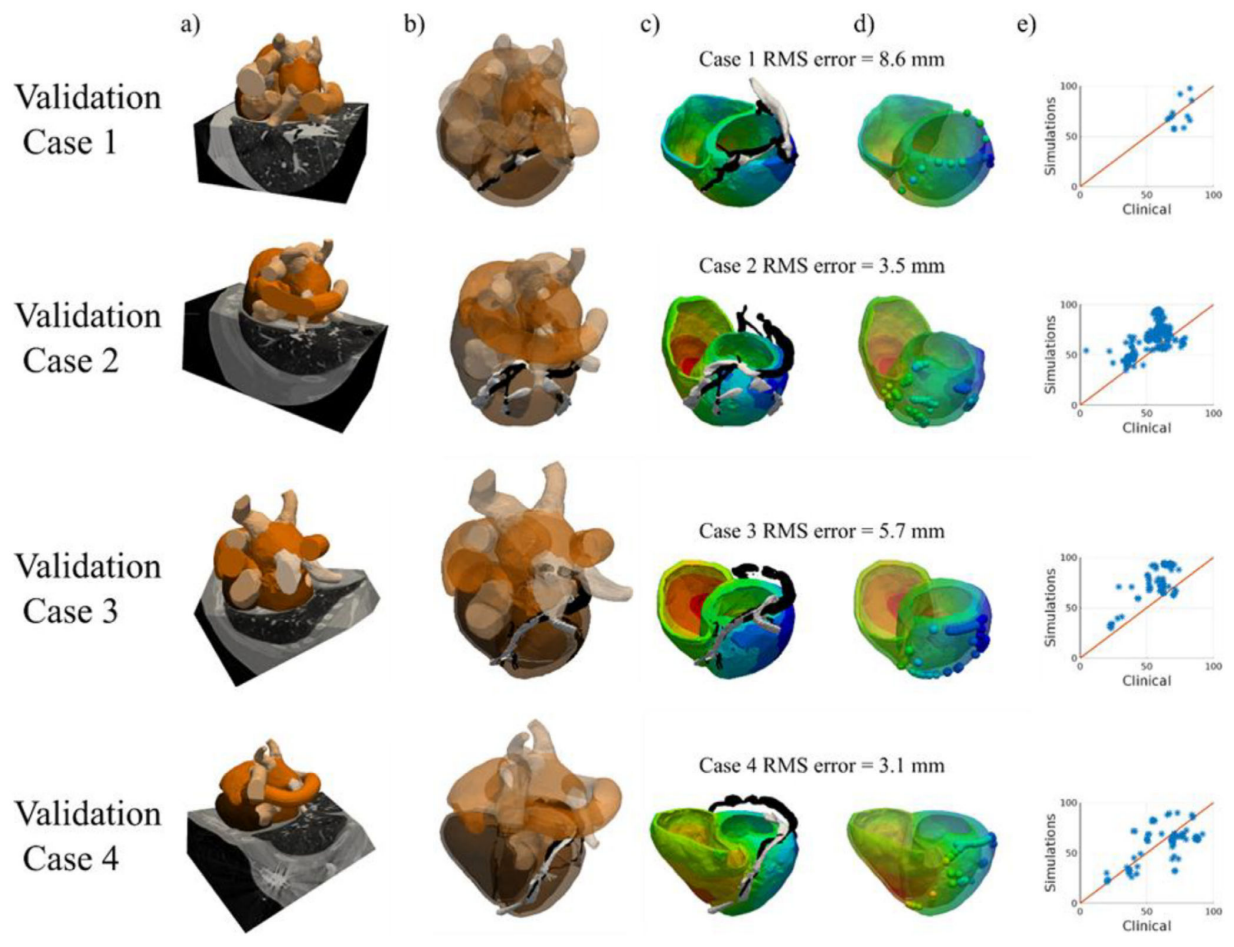
(a) CT images were acquired for 4 CRT upgrade patients and were automatically segmented using the Philips model based tool. (b) The EAM from Ensite NavX (white) was mapped to the coronary sinus semi-automatically segmented from the CT images (black). (c) The electrical activation across the ventricles from RV pacing was simulated. (d) The EAM local activation times were mapped onto the model (spheres) and the RMS distance error of the mapped EAM sites onto the models is stated for each case. (e) The LAT as a percentage of the QRS duration was compared between the clinical measurements and the model simulations with a six-fold increased conduction velocity in the ventricular endocardium.

A set of 4 CT patient cases was used for cross-modality validation of the rule based material property models (normal, with anterior or posterior block, with slow septal conduction and with fast endocardial conduction) (Fig. 5). No scar data was segmented, so the scar model was not tested with these CT data sets.

**Fig. 6:**
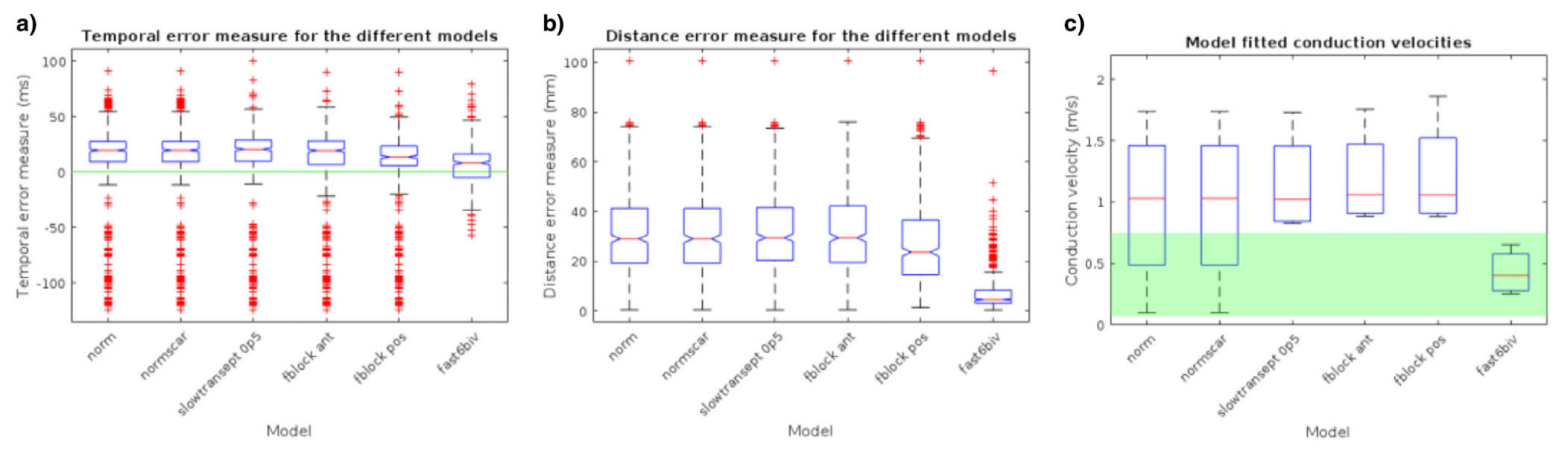
Simulations were run for the 4 CT cases, with a basic model, slow septum, fast endocardial conduction, anterior or posterior functional block. Boxplots of the (a) temporal error, (b) distance error, and (c) conduction velocities are shown for each model. The conduction velocities which are between the physiologically plausible range (0.07–0.75 m/s) are highlighted in green.

The temporal and distance error measures were calculated for the six electrophysiology models (Fig. 6a and b). One-way ANOVA indicated that there were no significant differences in the mean temporal errors (*p*-value > 0.05), while there were found to be significant differences in the mean distance errors of the six electrophysiology models (*p*-value < 0.001). Tukey post-hoc tests indicated that six-fold fast endocardial conduction (7.5 ± 0.7 mm) had a significantly improved mean distance error measure compared to the other models (Normal: 31.4 ± 0.7 mm; with anterior (32.2 ± 0.7 mm) or posterior block (27.5 ± 0.7 mm), and with slow septal conduction (32.0 ± 0.7 mm). The model fitted conduction velocities were within physiologically plausible ranges (0.07–0.75 m/s) for the 4 CT data sets (0.43 ± 0.18 m/s) only for the fast endocardial conduction models (Fig. 6c).

## 5 Discussion

In this study we developed a model that robustly simulates the electrical activation across the heart using non-invasive data. Rule-based methods for defining the electrical properties of the heart were investigated for 14 CMR cases and an additional 4 CT patient cases. The importance of the inclusion of scar, functional block, slow septal conduction and fast endocardial conduction in simulating the electrical activity across the heart was studied.

Our results indicate that fast endocardial conduction is the most important factor in constraining the model to physiologically plausible parameters consistent with experimental studies (latest electrical activation in a non-septal region with conduction velocities of 0.07–0.75 m/s) and resulting in sub-10 mm errors on average. This mean distance error needs to be considered in the clinical context. For CRT pacing lead optimization, the activation patterns needs to identify the optimal AHA segment. Endocardially and epicardially each AHA segment in the eighteen models had a mean size of 31.0 ± 4.6 mm x 14.4 ± 1.4 mm and 45.2 ± 4.3 mm x 30.2 ± 4.0 mm, respectively. The mean distance errors of the CMR cases (9.2 ± 0.5 mm) and the CT cases (7.5 ± 0.7 mm) are well within the sensitivity to identify the optimal AHA segment.

The underlying mechanism behind fast endocardial conduction is still unknown, with either retrograde activation of the Purkinje network or intrinsic properties of the endocardial layer hypothesized as reasons for an increase in the endocardial conductivity (Akar et al., 2004; Berruezo et al., 2004; Hyde et al., 2015; Strik et al., 2012; Taccardi et al., 2008). Experimental studies have found a transmural gradient in cellular properties across the ventricles, with increases in the cell areas (Stoker et al., 1982), gap junction density (Glukhov et al., 2012), and sodium channel densities (Gaborit et al., 2007) corresponding to increased conduction velocity in the endocardium in comparison to the epicardium. However, these experimentally observed changes in the endocardial cells are unable to fully explain the reported 1.5–4-fold increase in the conduction velocity (Draper and Mya-Tu, 1959; Myerburg et al., 1978; Strik et al., 2011).

An alternative explanation for fast endocardial conduction is retrograde activation of the Purkinje network, which has been observed during RV pacing (Damato et al., 1970). In experimental studies, Purkinje fibers arborizations have primarily been found in the lower third of the ventricles (Myerburg et al., 1972; Spach et al., 1963), though endocardial breakthrough sites have also been identified further up near the base of the left ventricle (Durrer et al., 1970). Simulations were run for fast endocardial conduction in the RV and the lower third of the LV, however it was found that the mean distance error had no significant improvements in comparison to the normal model, regardless of the increase in endocardial conduction velocity, unlike the models with a fast endocardial layer extending fully up the ventricles where the mean distant error improved as the fast endocardial conduction ration increased (Appendix B).

To date, there is a lack of evidence to clearly differentiate between the two mechanisms for fast endocardial conduction. In animal studies, the Purkinje network has been found to have a three to six fold increase in the conduction velocity in comparison to the myocardial tissue (Draper and Mya-Tu, 1959; Myerburg et al., 1978; Sano et al., 1959), while the superficial layers of the sub-endocardial ventricular muscle was found to have a 1.5–4 fold increase (Draper and Mya-Tu, 1959; Myerburg et al., 1978; Strik et al., 2011). Our simulations were also unable to distiguish between the two mechanisms, with five-fold to ten-fold increases in the fast endocardial conduction yielding similar temporal and distance errors for models where the fast endocardial conduction layer was defined as extending from the apex to base of the ventricles.

Other simulation studies of cardiac electrophysiology have specifically included in a Purkinje network represented as a 1D cable network (Romero et al., 2010; Vigmond and Stuyvers, 2016) or as activation sites (Kerckhoffs et al., 2012; Tobon-Gomez et al., 2013) based on experimental studies (Durrer et al., 1970; Usyk et al., 2002). The Purkinje system anatomy is individual specific, however in-vivo Purkinje fibers are unable to be imaged with current methods. Electrophysiology simulation studies have used manually delineated models of the Purkinje network (Romero et al., 2010) or randomly generating activation onset sites on the endocardial surface (Tobon-Gomez et al., 2013), while patient-specific modeling of the Purkinje network has relied on complex iterative methods and invasive electro-anatomical data (Cárdenes et al., 2015; Vergara et al., 2014). The aim of this study was to investigate simple rule-based methods that can be parameterized from non-invasive clinical measurements, so we have assumed that a fast endocardial layer approximates either the Purkinje network or the rapid conduction of the sub-endocardial layer. Further investigation into the importance of Purkinje network activation in comparison to this approximation remains to be done.

In contrast to clinical studies where the scar plays a significant role in response to CRT, our study has shown that scar was not the main determinant of activation times. However, the conclusions in this study need to be taken in the context of the aims of this paper, which were to investigate the relative importance of various factors effecting the electrical substrate of the ventricles. Scar, in addition to its effects on the electrical properties, has also been found to effect the mechanical properties of the cardiac tissues, with a higher density of collagen fibers in scarred tissue leading to an increase in the mechanical stiffness and decreased contractility (Jugdutt et al., 1996). Although in this study, we have found that scar is not important, it is in the context of the electrophysiology pattern, while the mechanical response of the heart is not modeled. In addition, in the study simulations a major effect of the scar would only be seen if it is completely transmural, otherwise the simulated electrical activation would simply go around the scar. So in a clinical context, it would be important to know where the scar is, but in the simulation of the electrical activation pattern, it is less important than including the fast endocardial conduction.

## 6 Limitations

The electrical activation in the heart has been shown to spread primarily along the fiber directions, so the myofiber orientations have a significant effect on the electrophysiology in the heart (Clerc, 1976; Roberts et al., 1979; Roth, 1997). In recent years, there has been increasing interest in personalizing the fiber orientation of the heart with in-vivo (Dou et al., 2003; Gamper et al., 2007; Geerts et al., 2002; Nguyen et al., 2014; Nielles-Vallespin et al., 2013; Toussaint et al., 2010, 2013; Wei et al., 2015) or ex-vivo (Helm et al., 2005; Holmes et al., 2000; Rohmer et al., 2007) diffusion tensor MRI (DTMRI). Though DTMRI is a promising technology for personalizing the fiber orientations in the heart, current challenges regarding the signal-to-noise ratio, long image acquisition times, low resolution and motion artifacts need to be overcome prior to using this method to personalize the fiber orientations in electrophysiology models of the heart. In previous computer modeling studies on rat (Bishop et al., 2009) and canine (Bayer et al., 2012) models, DTMRI-based and rule-based methods for determining the fiber orientation yielded similar electrical activation patterns. In this manuscript, we have thus assumed that the fiber orientations can be described adequately with rule-based methods.

Activation time measurements were only recorded on the free wall of the LV epicardium from within the coronary veins. This places inherent limitations on the locations that activation times can be measured. To confirm that measurements were made from across the epicardium, we have mapped recording sites onto an AHA plot (see Appendix A) and found multiple measurements points from all LV epicardium regions. The model was therefore only evaluated against epicardial activation time measurements. While complete activation time mapping would be ideal, patient measurements must be clinically tractable. We focused our measurements on the epicardium as this is the location of conventional CRT pacing and we would like to predict the latest epicardial activation time, using simulations, to identify potential optimal CRT pacing lead locations.

Another limitation of the models is that the regions of the heart were categorized into normal bulk myocardium, scar, functional block, slow septal conduction and fast endocardial conduction layers, with the electrical modeling parameters assumed to be homogeneous within each region. The conduction velocity in each region was set to be zero (scar or functional block) or scaled to be relative to the bulk myocardial tissue. However, regional heterogeneities have been found even across normal cardiac tissue, with varying properties across the heart transmurally, apical-basally and between the ventricles. In addition the infarcted regions were segmented based on MRI images, with no consideration of the heterogeneities across the border zone of the scar data. These additional heterogeneities could affect the conduction velocities across the heart. However, the degree of heterogeneity in the cardiac substrate is still unknown, particularly in diseased hearts. The myocardial conduction velocity was parameterized based on the QRS duration, a singular measure from the non-invasive 12 lead ECG traces. Other information can be derived from the ECG traces, such as the electrical axis which describes the main direction of the electrical depolarization in the heart. This information could potentially be used to further personalize the non-bulk myocardial conductivities in the heart model, such as differentiation of the RV and LV endocardial conduction velocities (Kayvanpour et al., 2015). However, the accuracy and robustness of these methods would still need to be investigated in future studies.

## 7 Clinical implications

In this study, we have developed and validated a novel approach to generate a realistic endocardial and epicardial electroanatomic activation map using only non-invasive modalities which are commonly used in standard clinical care (CMR/CT and 12-lead ECG). This approach was shown to work across 2 imaging modalities (CMR/CT). As only non-invasive data is required to predict the electrical activation of the ventricles, these simulations can be run prior to CRT implantation. In contrast to previous work relying on bidomain or monodomain models, our simulations used Eikonal equations to approximate the spread of the electrical activation across the heart, which requires a fraction of the computational costs (Neic et al., 2017). In this study, each simulation of the heart electrical activation took < 30 s on a standard desktop computer. While body surface potential mapping is a commercially available non-invasive technique that can be also be used to map the electrical activation of the heart, its cost remains a barrier to widespread clinical adoption. Our method presented here is fast, cheap and uses non-invasive clinical images and data acquired as part of standard clinical practice allowing for easy integration into standard clinical workflows to prospectively predict the latest point of electrical activation in the heart prior to device implantation for CRT patients.

## 8 Conclusion

The combination of a rule-based method for the fiber orientation (Bayer et al., 2012) with a rule-based description of the heterogeneous material properties of the electrical system in the heart allows for electrophysiology models to be developed with minimal non-invasive data (medical images such as CT or MRI and 12 lead ECG to determine the QRS duration) prior to device implantation. There is evidence that the optimal LV pacing site for CRT lies in the latest electrically activating region (Zanon et al., 2014). A model that incorporates a fast endocardial conduction layer is able predict the electrical activation to sub-10 mm accuracy. These model can potentially be a useful clinical planning tool to prospectively predict the latest point of electrical activation in the heart prior to device implantation (Table A9).

## Supplementary Material

Appendices

## Abbreviations

CRT: cardiac resynchronization therapy
LBBB: left bundle branch block
LV: left ventricle
RV: right ventricle
EAM: electroanatomical mapping
CECMR: contrast enhanced cardiac magnetic resonance
LAT: local activation time
CS: coronary sinus
DT MRI: diffusion tensor magnetic resonance imaging

## References

[R1] Aguado-Sierra J, Krishnamurthy A, Villongco C, Chuang J, Howard E, Gonzales MJ, Omens J, Krummen DE, Narayan S, Kerckhoffs RC (2011). Kerckhoffs RC Patient-specific modeling of dyssynchronous heart failure: a case study. Prog Biophys Mol Biol.

[R2] Akar FG, Spragg DD, Tunin RS, Kass DA, Tomaselli GF (2004). Mechanisms underlying conduction slowing and arrhythmogenesis in nonischemic dilated cardiomyopathy. Circ Res.

[R3] Auricchio A, Fantoni C, Regoli F, Carbucicchio C, Goette A, Geller C, Kloss M, Klein H (2004). Characterization of left ventricular activation in patients with heart failure and left bundle-branch block. Circulation.

[R4] Bayer JD, Blake RC, Plank G, Trayanova NA (2012). A novel rule-based algorithm for assigning myocardial fiber orientation to computational heart models. Ann Biomed Eng.

[R5] Berruezo A, Mont L, Nava S, Chueca E, Bartholomay E, Brugada J (2004). Electrocardiographic recognition of the epicardial origin of ventricular tachycardias. Circulation.

[R6] Bishop MJ, Hales P, Plank G, Gavaghan DJ, Scheider J, Grau V (2009). Comparison of rule-based and DTMRI-derived fibre architecture in a whole rat ventricular computational model.

[R7] Cárdenes R, Sebastian R, Soto-Iglesias D, Berruezo A, Camara O (2015). Estimation of Purkinje trees from electro-anatomical mapping of the left ventricle using minimal cost geodesics. Med Image Anal.

[R8] Clerc L (1976). Directional differences of impulse spread in trabecular muscle from mammalian heart. J Physiol (Lond).

[R9] Crozier A, Blazevic B, Lamata P, Plank G, Ginks M, Duckett S, Sohal M, Shetty A, Rinaldi CA, Razavi R, Smith NP (2015). The relative role of patient physiology and device optimisation in cardiac resynchronisation therapy: a computational modelling study. J Mol Cell Cardiol.

[R10] Damato AN, Lau SH, Bobb GA (1970). Studies on ventriculo-atrial conduction and the reentry phenomenon. Circulation.

[R11] de Bakker JM, van Capelle FJ, Janse MJ, Tasseron S, Vermeulen JT, de Jonge N, Lahpor JR (1993). Slow conduction in the infarcted human heart. ’Zigzag’ course of activation. Circulation.

[R12] Derval N, Bordachar P, Lim HS, Sacher F, Ploux S, Laborderie J, Steendijk P, Deplagne A, Ritter P, Garrigue S (2014). Impact of pacing site on QRS duration and its relationship to hemodynamic response in cardiac resynchronization therapy for congestive heart failure. J Cardiovasc Electrophysiol.

[R13] Dou J, Tseng WYI, Reese TG, Wedeen VJ (2003). Combined diffusion and strain MRI reveals structure and function of human myocardial laminar sheets in vivo. Magn Reson Med.

[R14] Draper M, Mya-Tu M (1959). A comparison of the conduction velocity in cardiac tissues of various mammals. Exp Physiol.

[R15] Durrer D, van Dam RT, Freud GE, Janse MJ, Meijler FL, Arzbaecher RC (1970). Total excitation of the isolated human heart. Circulation.

[R16] Gaborit N, Le Bouter S, Szuts V, Varro A, Escande D, Nattel S, Demolombe S (2007). Regional and tissue specific transcript signatures of ion channel genes in the non-diseased human heart. J Physiol (Lond).

[R17] Gamper U, Boesiger P, Kozerke S (2007). Diffusion imaging of the in vivo heart using spin echoes-considerations on bulk motion sensitivity. Magn Reson Med.

[R18] Geerts L, Bovendeerd P, Nicolay K, Arts T (2002). Characterization of the normal cardiac myofiber field in goat measured with MR-diffusion tensor imaging. Am J Physiol Heart Circulat Physiol.

[R19] Giffard-Roisin S, Jackson T, Fovargue L, Lee J, Delingette H, Razavi R, Ayache N, Sermesant M (2017). Noninvasive personalization of a cardiac electrophysiology model from body surface potential mapping. IEEE Trans Biomed Eng.

[R20] Glukhov AV, Fedorov VV, Kalish PW, Ravikumar VK, Lou Q, Janks D, Schuessler RB, Moazami N, Efimov IR (2012). Conduction remodeling in human end-stage non-ischemic left ventricular cardiomyopathy. Circulation Circulationaha.

[R21] Helm PA, Tseng HJ, Younes L, McVeigh ER, Winslow RL (2005). Ex vivo 3D diffusion tensor imaging and quantification of cardiac laminar structure. Magn Reson Med.

[R22] Holmes AA, Scollan D, Winslow RL (2000). Direct histological validation of diffusion tensor MRI in formaldehyde-fixed myocardium. Magn Reson Med.

[R23] Hyde ER, Behar JM, Claridge S, Jackson T, Lee AW, Remme EW, Sohal M, Plank G, Razavi R, Rinaldi CA, Niederer SA (2015). Beneficial effect on cardiac resynchronization from left ventricular endocardial pacing is mediated by early access to high conduction velocity tissue: electrophysiological simulation study. Circ Arrhythm Electrophysiol.

[R24] Jia P, Ramanathan C, Ghanem RN, Ryu K, Varma N, Rudy Y (2006). Electrocardiographic imaging of cardiac resynchronization therapy in heart failure: observation of variable electrophysiologic responses. Heart Rhythm.

[R25] Jugdutt BI, Joljart MJ, Khan MI (1996). Rate of collagen deposition during healing and ventricular remodeling after myocardial infarction in rat and dog models. Circulation.

[R26] Kayvanpour E, Mansi T, Sedaghat-Hamedani F, Amr A, Neumann D, Georgescu B, Seegerer P, Kamen A, Haas J, Frese KS (2015). Towards personalized cardiology: multi-scale modeling of the failing heart. PLoS ONE.

[R27] Kerckhoffs RC, Omens JH, McCulloch AD (2012). Mechanical discoordination increases continuously after the onset of left bundle branch block despite constant electrical dyssynchrony in a computational model of cardiac electromechanics and growth. Europace.

[R28] Khan FZ, Virdee MS, Fynn SP, Dutka DP (2009). Left ventricular lead placement in cardiac resynchronization therapy: where and how?. Europace.

[R29] Khan FZ, Virdee MS, Palmer CR, Pugh PJ, O’Halloran D, Elsik M, Read PA, Begley D, Fynn SP, Dutka DP (2012). Targeted left ventricular lead placement to guide cardiac resynchronization therapy: the TARGET study: a randomized, controlled trial. J Am Coll Cardiol.

[R30] Kleber AG, Rudy Y (2004). Basic mechanisms of cardiac impulse propagation and associated arrhythmias. Physiol Rev.

[R31] Lee AW, Crozier A, Hyde ER, Lamata P, Truong M, Sohal M, Jackson T, Behar JM, Claridge S, Shetty A, Sammut E (2017). Biophysical modeling to determine the optimization of left ventricular pacing site and AV/VV delays in the acute and chronic phase of cardiac resynchronization therapy. J Cardiovasc Electrophysiol.

[R32] Leyva F, Foley PW, Chalil S, Ratib K, Smith RE, Prinzen F, Auricchio A (2011). Cardiac resynchronization therapy guided by late gadolinium-enhancement cardiovascular magnetic resonance. J Cardiovasc Magnet Resonance.

[R33] Mafi Rad M, Blaauw Y, Dinh T, Pison L, Crijns HJ, Prinzen FW, Vernooy K (2014). Different regions of latest electrical activation during left bundle-branch block and right ventricular pacing in cardiac resynchronization therapy patients determined by coronary venous electro-anatomic mapping. Eur J Heart Fail.

[R34] Mafi Rad M, Blaauw Y, Dinh T, Pison L, Crijns HJ, Prinzen FW, Vernooy K (2014). Left ventricular lead placement in the latest activated region guided by coronary venous electroanatomic mapping. Ep Europace.

[R35] Miri R, Reumann M, Farina D, Dossel O (2009). Concurrent optimization of timing delays and electrode positioning in biventricular pacing based on a computer heart model assuming 17 left ventricular segments. Biomedizinische Technik.

[R36] Mountney P, Behar JM, Toth D, Panayiotou M, Reiml S, Jolly M-P, Karim R, Zhang L, Brost A, Rinaldi CA (2017). A planning and guidance platform for cardiac resynchronization therapy. IEEE Trans Med Imaging.

[R37] Myerburg RJ, Gelband H, Nilsson K, Castellanos A, Morales AR, Bassett AL (1978). The role of canine superficial ventricular muscle fibers in endocardial impulse distribution. Circ Res.

[R38] Myerburg RJ, Nilsson K, Gelband H (1972). Physiology of canine intraventricular conduction and endocardial excitation. Circ Res.

[R39] Neic A, Campos FO, Prassl AJ, Niederer SA, Bishop MJ, Vigmond EJ, Plank G (2017). Efficient computation of electrograms and ECGs in human whole heart simulations using a reaction-eikonal model. J Comput Phys.

[R40] Nguyen C, Fan Z, Sharif B, He Y, Dharmakumar R, Berman DS, Li D (2014). In vivo three-dimensional high resolution cardiac diffusion-weighted MRI: a motion compensated diffusion-prepared balanced steady-state free precession approach. Magn Reson Med.

[R41] Nguyen UC, Mafi-Rad M, Aben JP, Smulders MW, Engels EB, van Stip-donk AM, Luermans JG, Bekkers SC, Prinzen FW, Vernooy K (2017). A novel approach for left ventricular lead placement in cardiac resynchronization therapy: intraprocedural integration of coronary venous electroanatomic mapping with delayed enhancement cardiac magnetic resonance imaging. Heart Rhythm.

[R42] Niederer S, Mitchell L, Smith N, Plank G (2011). Simulating human cardiac electrophysiology on clinical time-scales. Front Physiol.

[R43] Nielles-Vallespin S, Mekkaoui C, Gatehouse P, Reese TG, Keegan J, Ferreira PF, Collins S, Speier P, Feiweier T, de Silva R, Jackowski MP (2013). In vivo diffusion tensor MRI of the human heart: reproducibility of breath-hold and navigator-based approaches. Magn Reson Med.

[R44] Okada JI, Washio T, Nakagawa M, Watanabe M, Kadooka Y, Kariya T, Yamashita H, Yamada Y, Momomura SI, Nagai R, Hisada T (2017). Multi-scale, tailor-made heart simulation can predict the effect of cardiac resynchronization therapy. J Mol Cell Cardiol.

[R45] Peters J, Ecabert O, Meyer C, Schramm H, Kneser R, Groth A, Weese J (2007). Automatic whole heart segmentation in static magnetic resonance image volumes. Medical Image Computing and Computer-Assisted Intervention-MICCAI 2007.

[R46] Peters NS, Green CR, Poole-Wilson PA, Severs NJ (1993). Reduced content of Connexin43 gap junctions in ventricular myocardium from hypertrophied and ischemic human hearts. Circulation.

[R47] Prinzen FW, Auricchio A (2008). Is echocardiographic assessment of dyssynchrony useful to select candidates for cardiac resynchronization therapy? Response to prinzen and Aurrichio: echocardiography is not useful before cardiac resynchronization therapy if QRS duration is available. Circulation.

[R48] Reumann M, Farina D, Miri R, Lurz S, Osswald B, Dossel O (2007a). Computer model for the optimization of AV and VV delay in cardiac resynchronization therapy. Med Biol Eng Comput.

[R49] Reumann M, Osswald B, Doessel O (2007b). Noninvasive, automatic optimization strategy in cardiac resynchronization therapy. Anatol J Cardiol Anadolu Kardiyoloji Dergisi.

[R50] Roberts DE, Hersh LT, Scher AM (1979). Influence of cardiac fiber orientation on wavefront voltage, conduction velocity, and tissue resistivity in the dog. Circ Res.

[R51] Rohmer D, Sitek A, Gullberg GT (2007). Reconstruction and visualization of fiber and laminar structure in the normal human heart from ex vivo diffusion tensor magnetic resonance imaging (DTMRI) data. Invest Radiol.

[R52] Romero D, Sebastian R, Bijnens BH, Zimmerman V, Boyle PM, Vigmond EJ, Frangi AF (2010). Effects of the Purkinje system and cardiac geometry on biventricular pacing: a model study. Ann Biomed Eng.

[R53] Roth BJ (1997). Electrical conductivity values used with the bidomain model of cardiac tissue. IEEE Trans Biomed Eng.

[R54] Saba S, Marek J, Schwartzman D, Jain S, Adelstein E, White P, Oyenuga OA, Onishi T, Soman P, Gorcsan J (2013). Echocardiography-guided left ventricular lead placement for cardiac resynchronization therapy clinical perspective: results of the speckle tracking assisted resynchronization therapy for electrode region trial. Circulation.

[R55] Sano T, Takayama N, Shimamoto T (1959). Directional difference of conduction velocity in the cardiac ventricular syncytium studied by microelectrodes. Circ Res.

[R56] Scollan DF, Holmes A, Winslow R, Forder J (1998). Histological validation of myocardial microstructure obtained from diffusion tensor magnetic resonance imaging. Am J Physiol Heart Circulat Physiol.

[R57] Seemann G, Keller D, Weiss D, Dossel O (2006). Modeling human ventricular geometry and fiber orientation based on diffusion tensor MRI. Computers in Cardiology.

[R58] Sermesant M, Chabiniok R, Chinchapatnam P, Mansi T, Billet F, Moireau P, Peyrat JM, Wong K, Relan J, Rhode K, Ginks M (2012). Patient-specific electromechanical models of the heart for the prediction of pacing acute effects in CRT: a preliminary clinical validation. Med Image Anal.

[R59] Singh JP, Klein HU, Huang DT, Reek S, Kuniss M, Quesada A, Barsheshet A, Cannom D, Goldenberg I, McNitt S, Daubert JP (2011). Left ventricular lead position and clinical outcome in the multicenter automatic defibrillator implantation trial-cardiac resynchronization therapy (MADIT-CRT) trial. Circulation.

[R60] Sohal M, Shetty A, Niederer S, Chen Z, Jackson T, Sammut E, Bostock J, Razavi R, Prinzen F, Rinaldi CA (2014). Delayed trans-septal activation results in comparable hemodynamic effect of left ventricular and biventricular endocardial pacing clinical perspective: insights from electroanatomical mapping. Circulation.

[R61] Sohal M, Shetty A, Niederer S, Lee A, Chen Z, Jackson T, Behar JM, Claridge S, Bostock J, Hyde E (2015). Mechanistic insights into the benefits of multisite pacing in cardiac resynchronization therapy: the importance of electrical substrate and rate of left ventricular activation. Heart Rhythm.

[R62] Spach MS, Huang S-n, Armstrong SI, Canent RV (1963). Demonstration of peripheral conduction system in human hearts. Circulation.

[R63] Stoker ME, Gerdes AM, May JF (1982). Regional differences in capillary density and myocyte size in the normal human heart. Anat Rec.

[R64] Strik M, Ploux S, Vernooy K, Prinzen FW (2011). Cardiac resynchronization therapy: refocus on the electrical substrate. Circ J.

[R65] Strik M, Rademakers LM, van Deursen CJ, van Hunnik A, Kuiper M, Klersy C, Auricchio A, Prinzen FW (2012). Endocardial left ventricular pacing improves cardiac resynchronization therapy in chronic asynchronous infarction and heart failure models. Circ Arrhythm Electrophysiol.

[R66] Strik M, van Deursen CJ, van Middendorp LB, van Hunnik A, Kuiper M, Auricchio A, Prinzen FW (2013). Transseptal conduction as an important determinant for cardiac resynchronization therapy, as revealed by extensive electrical mapping in the dyssynchronous canine heart. Circ Arrhythm Electrophysiol.

[R67] Taccardi B, Punske BB, Macchi E, MacLeod RS, Ershler PR (2008). Epicardial and intramural excitation during ventricular pacing: effect of myocardial structure. Am J Physiol Heart Circulat Physiol.

[R68] Tobon-Gomez C, Duchateau N, Sebastian R, Marchesseau S, Camara O, Donal E, De Craene M, Pashaei A, Relan J, Steghofer M (2013). Understanding the mechanisms amenable to CRT response: from pre-operative multimodal image data to patient-specific computational models. Med Biol Eng Comput.

[R69] Toussaint N, Sermesant M, Stoeck C, Kozerke S, Batchelor P (2010). In vivo human 3D cardiac fibre architecture: reconstruction using curvilinear interpolation of diffusion tensor images. Medical Image Computing and Computer-Assisted Intervention?.

[R70] Toussaint N, Stoeck CT, Schaeffter T, Kozerke S, Sermesant M, Batchelor PG (2013). In vivo human cardiac fibre architecture estimation using shape-based diffusion tensor processing. Med Image Anal.

[R71] Usyk TP, LeGrice IJ, McCulloch AD (2002). Computational model of three-dimensional cardiac electromechanics. Comput Vis Sci.

[R72] Vergara C, Palamara S, Catanzariti D, Nobile F, Faggiano E, Pangrazzi C, Centonze M, Maines M, Quarteroni A, Vergara G (2014). Patient-specific generation of the Purkinje network driven by clinical measurements of a normal propagation. Med Biol Eng Comput.

[R73] Vigmond EJ, Hughes M, Plank G, Leon LJ (2003). Computational tools for modeling electrical activity in cardiac tissue. J Electrocardiol.

[R74] Vigmond EJ, Stuyvers BD (2016). Modeling our understanding of the his-Purkinje system. Prog Biophys Mol Biol.

[R75] Villongco CT, Krummen DE, Omens JH, McCulloch AD (2016). Non-invasive, model-based measures of ventricular electrical dyssynchrony for predicting CRT outcomes. Europace.

[R76] Wei H, Viallon M, Delattre BM, Moulin K, Yang F, Croisille P, Zhu Y (2015). Free-breathing diffusion tensor imaging and tractography of the human heart in healthy volunteers using wavelet-based image fusion. IEEE Trans Med Imaging.

[R77] Weidmann S (1970). Electrical constants of trabecular muscle from mammalian heart. J Physiol (Lond).

[R78] Ypenburg C, van Bommel RJ, Delgado V, Mollema Sa, Bleeker GB, Boersma E, Schalij MJ, Bax JJ (2008). Optimal left ventricular lead position predicts reverse remodeling and survival after cardiac resynchronization therapy. J Am Coll Cardiol.

[R79] Zanon F, Baracca E, Pastore G, Fraccaro C, Roncon L, Aggio S, Noventa F, Mazza A, Prinzen F (2014). Determination of the longest intrapatient left ventricular electrical delay may predict acute hemodynamic improvement in patients after cardiac resynchronization therapy. Circ Arrhythm Electrophysiol.

