# Supplementary material for: A rule-based method for predicting the electrical activation of the heart with cardiac resynchronization therapy from non-invasive clinical data
Source: Med Image Anal. Author manuscript; Available in PMC 2019 Sep 16. (PMC6746621; doi:10.1016/j.media.2019.06.017)
Supplement: Appendices [file EMS84284-supplement-Appendices.pdf]

Appendix A

Activation patterns were only measured in the coronary veins. This potentially limited the locations where activations times were measured. To quantify the distribution of measurement locations all measurement sites from the 14 CMR cases were projected onto the AHA maps Fig. A7. This shows that measurements are acquired from all LV free wall regions.

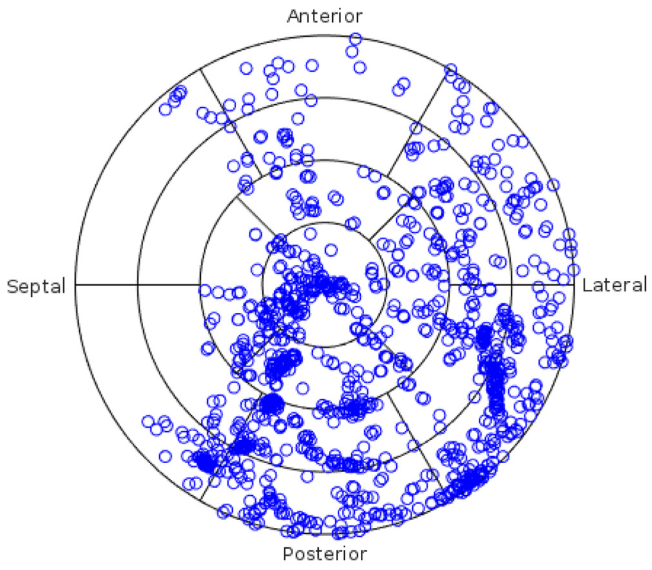

Fig. A7. AHA map of the distribution of the sites on the LV wall where the LAT was measured from RV paced sites.

Appendix B. Investigating the sensitivity of the distance error to changes in the anatomy, fibres, anisotropy ratio and the slow septal conductivity ratio

Six electrophysiological rule-based electrophysiological models were used to simulate the electrical activation of the heart: Normal (norm), inclusive of scar (normscar), inclusive of slow septal conductivity (slowtransept0p5), inclusive of anterior (fblock ant) or posterior functional block (fblock pos), inclusive of fast endocardial conduction (fast6biv).

The sensitivity of the distance errors to the parameters used to describe these models was investigated. One-way ANOVA was used to find any statistically significant differences (p-value$<$0.001) in the mean distance error when the parameters, such as anatomical features, fibre orientations, anisotropy ratio and slow septal conductivity ratios were changed. Tukey post-hoc tests were then used to identify where a significant difference exists. In all cases, the same conclusion was reached: that fast endocardial conduction improved the accuracy of the model simulations in terms of the distance error measure compared to the other models.

Results are presented in graphs in the following sections:

a) Box plots of the distance errors for each model. The edges of each blue box represent the 1st and 3rd quantiles, central mark represents the median, with the whiskers extending to the most extreme data points that are not considered outliers. The red crosses represent the outliers.
b) Plots of the mean estimates and comparison intervals between each model. The mean of each model is represented with a circle, with the comparison interval represented as the line. The blue line in each plot is the selected model that is being compared to the rest of the models, with red lines representing

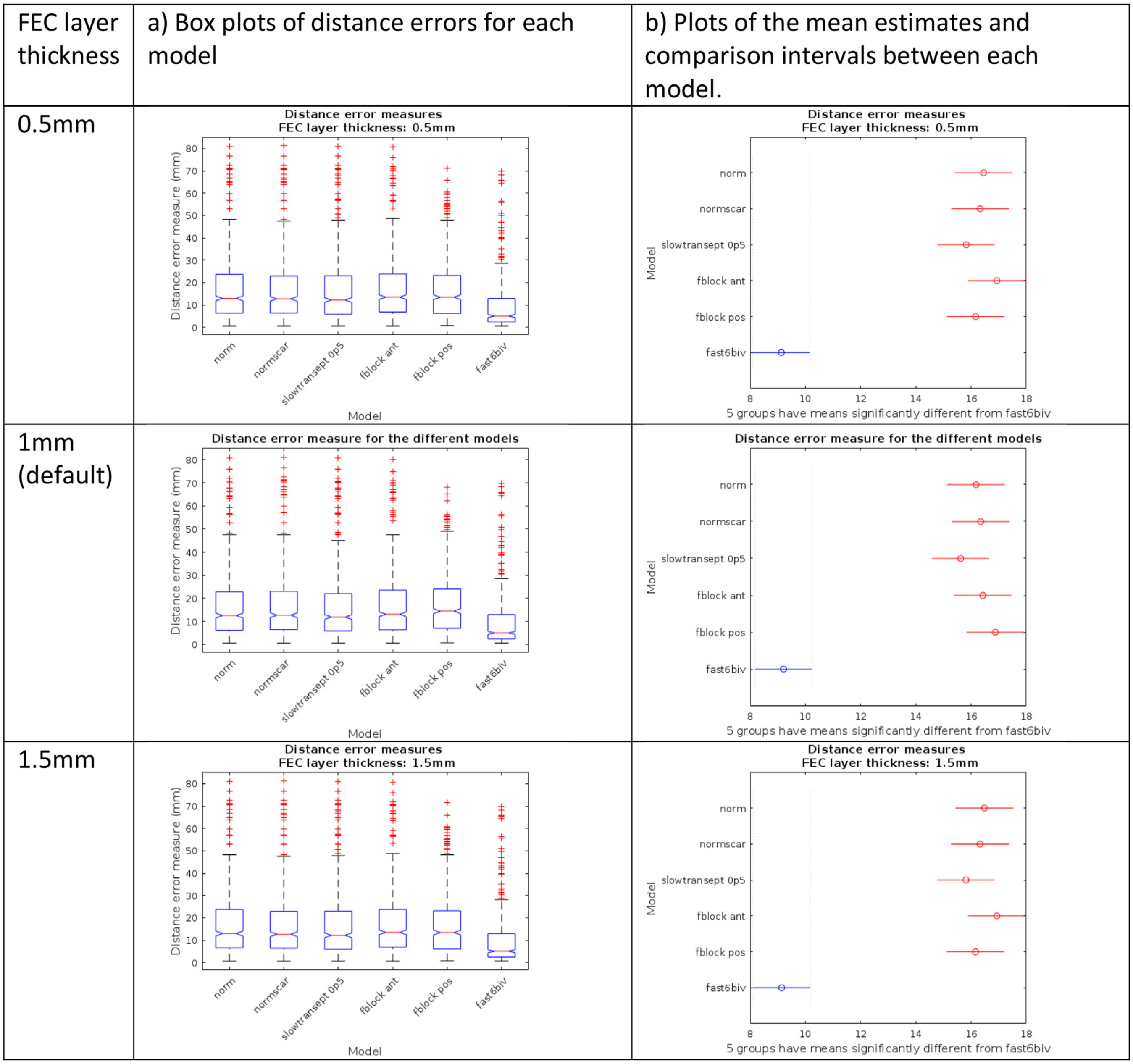

Fig. A8. Plots showing the sensitivity to changes in the fast endocardial conduction layer thickness for the different models (Normal: norm, inclusive of scar: normscar, inclusive of slow septal conductivity: slowtransept0p5, inclusive of anterior: fblock ant or posterior functional block: fblock pos, inclusive of fast endocardial conduction: fast6biv).

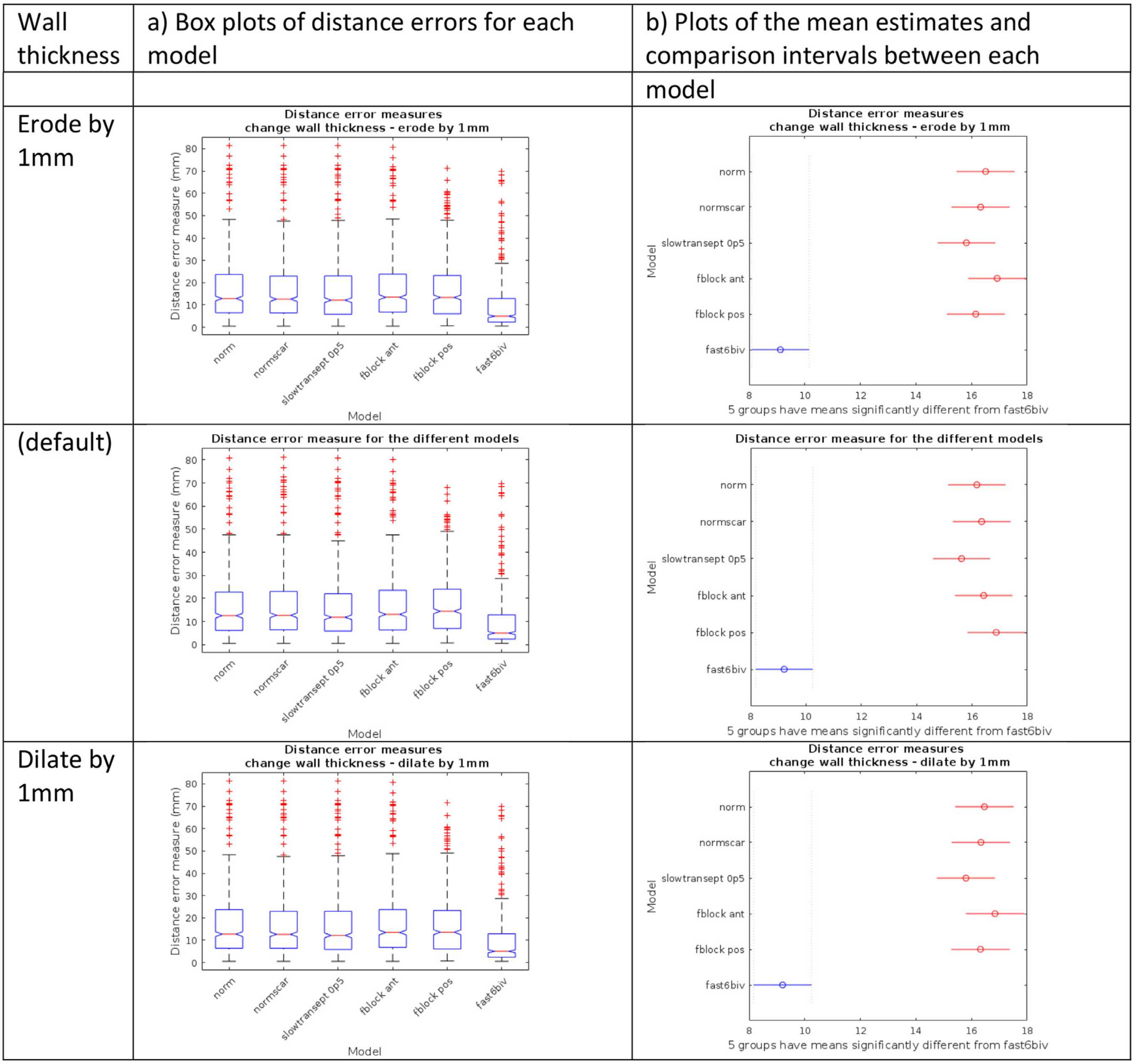

Fig. A9. Plots showing the sensitivity to changes in wall thickness for the different models (Normal: norm, inclusive of scar: normscar, inclusive of slow septal conductivity: slowtransept0p5, inclusive of anterior functional block: fblock ant or posterior functional block: fblock pos, inclusive of fast endocardial conduction: fast6biv).

models that are significantly different, and grey lines indicating models that have no significant differences with the selected model.

B1. Anatomy

The sensitivity of the distance error measures to change in the thickness of the fast endocardial conduction (FEC) layer (subject to 0.5 mm dilation or erosion) (Fig. A8) or change in the wall thickness (subject to 1 mm dilation or erosion) (Fig. A9) was minimal across all the models <1 mm difference in the mean distance errors for the models (Tables A1 & A2).

B2. Fast endocardial conduction ratio

The fast endocardial conduction (FEC) model was originally defined with a thin layer on the endocardial surface having a 6-fold conduction velocity compared to the bulk myocardial conduction velocity. The sensitivity of the distance error measures to this ratio was investigated for ratios ranging from 1-fold (normal model) to 10-fold (fast10biv) (Fig. A10). One-way ANOVA found that were significant differences between the different models and Tukey post-hoc tests found that as the FEC ratio increased, the distance error gradually improved. It was also found that there were no significant differences in the distance error between 5-fold to 10-fold increases in the FEC ratio (Table A3).

B3. Bottom third fast endocardial conduction models

In addition to the FEC simulations in the manuscript where the FEC layer was set as extending from apex to base (all RV and LV endocardium), simulations were also run for models where only the bottom third of the LV endocardium and all of the RV en-

Table A1
Mean distance errors with changes in the fast endocardial conduction (FEC) layer thickness for the different models (Normal: norm, inclusive of scar: normscar, inclusive of slow septal conductivity: slowtransept0p5, inclusive of anterior: fblock ant or posterior functional block: fblock pos, inclusive of fast endocardial conduction: fast6biv). .

FEC		Norm	Scar	slowsept	fblock ant	fblock pos	fast6biv
0.5 mm	Mean	16.45	16.33	15.82	16.93	16.17	9.13
	Std error	0.52	0.52	0.51	0.52	0.52	0.51
1.0 mm	Mean	16.18	16.35	15.62	16.42	16.88	9.21
	Std error	0.52	0.52	0.51	0.51	0.51	0.51
1.5 mm	Mean	16.48	16.32	15.81	16.93	16.16	9.14
	Std error	0.52	0.52	0.52	0.52	0.52	0.52

Table A2
Mean distance errors with changes in the wall thickness for the different models (normal: norm, inclusive of scar: normscar, inclusive of slow septal conductivity: slowsept, inclusive of anterior functional block: fblock ant or posterior functional block: fblock pos, inclusive of fast endocardial conduction: fast6biv).

Wall thickness		Norm	Scar	Slowsept	fblock ant	fblock pos	fast6biv
Erode by 1mm	Mean	16.51	16.33	15.82	16.92	16.15	9.12
	Std error	0.52	0.52	0.51	0.52	0.52	0.51
Default	Mean	16.18	16.35	15.62	16.42	16.88	9.21
	Std error	0.52	0.52	0.51	0.51	0.51	0.51
Dilate by 1mm	Mean	16.46	16.34	15.8	16.84	16.32	9.2
	Std error	0.52	0.52	0.52	0.52	0.52	0.52

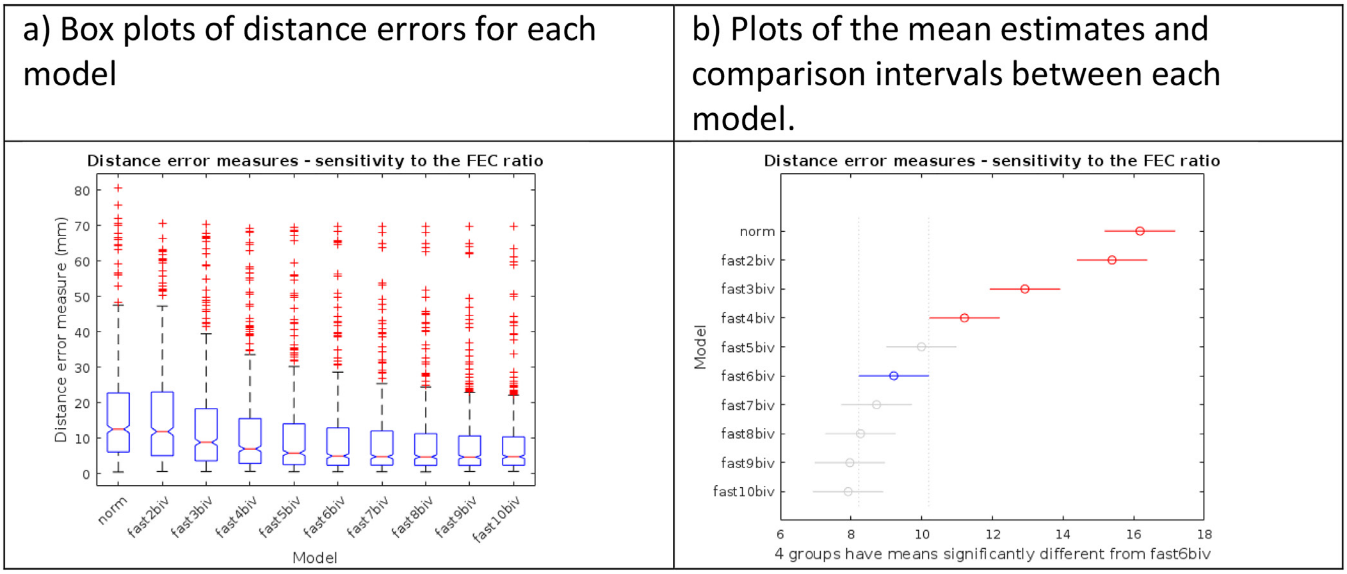

Fig. A10. Plots showing the sensitivity to changes in the fast endocardial conduction velocity ratio ranging from 1-fold (norm) to 10-fold (fast10biv).

Table A3
Mean distance errors with changes in the fast endocardial conduction (FEC) ratio ranging from 1-fold (norm) to 10-fold (FEC10).

Model	Norm	FEC2	FEC3	FEC4	FEC5	FEC6	FEC7	FEC8	FEC9	fast6biv
Mean	16.18	15.39	12.92	11.21	9.99	9.21	8.72	8.27	7.97	7.92
Std error	0.45	0.45	0.45	0.44	0.44	0.44	0.45	0.44	0.44	0.44

docardium have increased conduction velocities in comparison to the bulk myocardium (Fig. A11). The increase in the FEC ratio was also varied, ranging from 1-fold (norm) to 10-fold (fast10 low3) (Fig. A12). One-way ANOVA found no significant differences between the mean distance errors between the models (p-value>0.1), with the mean values ranging from 15.1–16.4 mm (Table A4).

B4. Fibre orientations

Fibre angles were initially defined using the Bayer et al. described fibre directions (1) An additional set of simulations were run with the Streeter et al. defined fibre directions (+/−60° across the myocardial wall (2). Normal, Scar, functional block in the anterior or posterior walls, slow septal conductivity and 6-fold fast endocardial conduction were analysed with the new fibre angle directions (Fig. A13). It was found that changes in the fibre orientations had little effect on the overall mean distance errors for the models (<1 mm difference) (Table A5).

B5. Anisotropy ratio

The anisotropy ratio for the conduction velocity across the fibres to along the fibres to was initially defined as 0.40:1.00 (3). The sensitivity of our model accuracy to this choice of anisotropy

Table A4
Mean distance errors with changes in the fast endocardial conduction (FEC) ratio ranging from 1-fold (norm) to 10-fold (FEC10), where the fast endocardial conduction layer extends over the RV endocardium and the bottom third of the LV endocardium.

Model	Norm	FEC2	FEC3	FEC6	FEC10
Mean	16.18	15.16	15.32	16.02	16.36
Std error	0.52	0.52	0.52	0.52	0.52

Table A5
Mean distance errors for changes in fibre rules orientations for the different models (normal: norm, inclusive of scar: scar, inclusive of slow septal conductivity: slowsept, inclusive of anterior: fblock ant or posterior functional block: fblock pos, inclusive of fast endocardial conduction: FEC6).

		Norm	Scar	Slowsept	fblock ant	fblock pos	FEC6
Bayer fibres	Mean	16.18	16.35	15.62	16.42	16.88	9.21
	Std error	0.52	0.52	0.51	0.51	0.51	0.51
Streeter fibres	Mean	16.14	16.34	15.64	16.54	16.03	9.14
	Std error	0.51	0.51	0.51	0.51	0.51	0.51

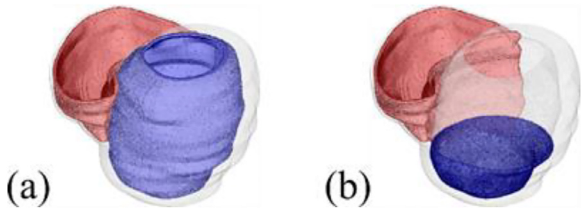

Fig. A11. Fast endocardial conduction models with increased conduction velocity in (a) the LV and RV endocardium and (b) all of the RV endocardium and the bottom third of the LV endocardium.

ratio was also investigated for anisotropy ratios: 0.16 (4) to 1.0 (no anisotropy) (Fig. A14). It was found that while there were significant differences in the mean distance error across the models for different anisotropy ratios, the conclusion that fast endocardial conduction was the most important factor remained consistent (Table A6).

B6. Slow septal conductivity

The transmural slow septal conduction velocity was initially set as 0.5 relative to the transmural conduction velocity of the bulk myocardial tissue. The sensitivity of the model accuracy to the slow transmural septal ratio was investigated, ranging from a ratio of 0.1 to 1.0 (normal) (Fig. A15). One-way ANOVA found that while no significant differences in the mean distance error was observed for transmural septal slowing ratios >0.15, the mean distance error increased significantly as the ratio reduced <0.15 (Table A7).

Similar conclusions were drawn for slow all septal conductivity model, the septum was slower both along the fibres and transverse to the fibre directions. The sensitivity of the model to the slow septal conduction ratio ranging from 0.1 to 1.0(normal) was investigated (Fig. A15). One-way ANOVA found that while no significant differences in the mean distance error was observed for transmural septal slowing ratios >0.5, the mean distance error increased significantly as the ratio reduced <0.5 (Table A8).

In the visualization of the meshes (Fig. A16), it was observed that as the slow transmural septal ratio< 0.15 or the slow all septal ratio <0.5, the latest point of electrical activation occurs in the septum. As we are interested in simulating patients with

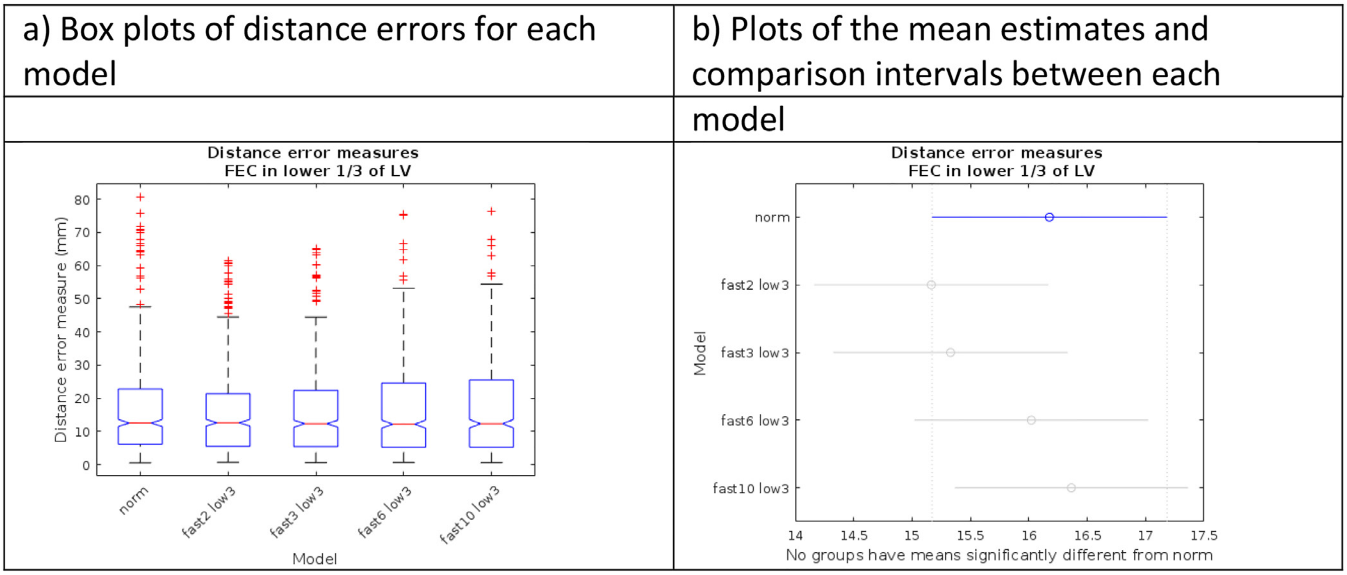

Fig. A12. Plots showing the sensitivity to changes in the fast endocardial conduction velocity ratio ranging from 1-fold (norm) to 10-fold (fast10 low3), where the fast endocardial conduction layer extends over the RV endocardium and the bottom third of the LV endocardium. .

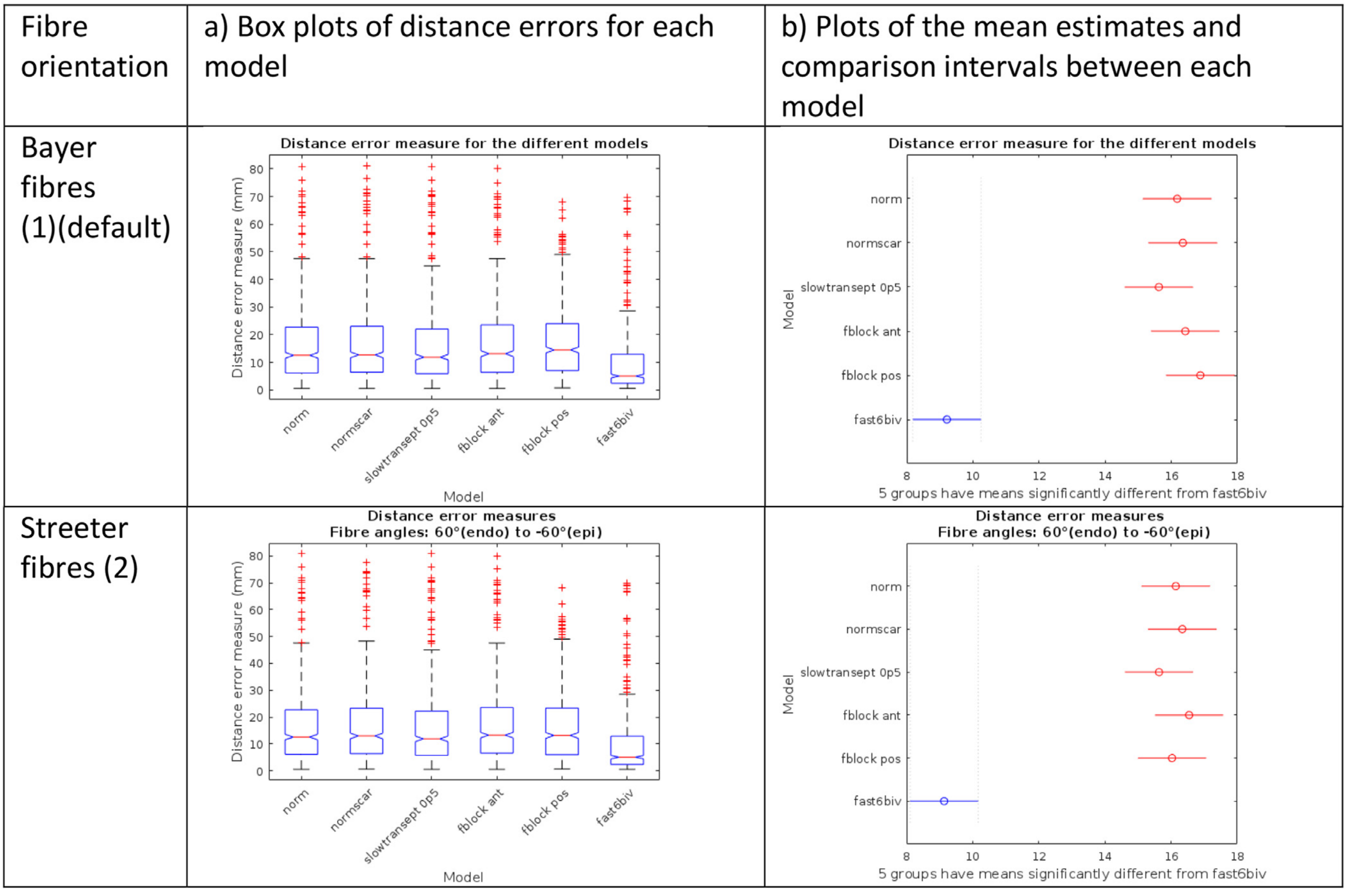

Fig. A13. Plots showing the sensitivity to changes in fibre orientation for the different models (Normal: norm, inclusive of scar: normscar, inclusive of slow septal conductivity: slowtransept0p5, inclusive of anterior: fblock ant or posterior functional block: fblock pos, inclusive of fast endocardial conduction: fast6biv).

Table A6
Mean distance errors for changes in anisotropy ratio for the conduction velocity transverse to the fibres: along the fibre directions ranging from 0.16:1.00 to 1.00:1.00 for the different models (normal: norm, inclusive of scar: scar, inclusive of slow septal conductivity: slowsept, inclusive of anterior: fblock ant or posterior functional block: fblock pos, inclusive of fast endocardial conduction: FEC6).

Anisotropy ratio		Norm	Scar	Slowsept	fblock ant	fblock pos	FEC6
0.16:1.00	Mean	13.69	12.81	12.97	14.94	13.76	9.85
	Std error	0.49	0.49	0.49	0.49	0.49	0.49
0.2:1.00	Mean	14.4	13.7	13.68	14.72	14.32	9.98
	Std error	0.5	0.5	0.5	0.5	0.5	0.5
0.4:1.00	Mean	16.18	16.35	15.62	16.42	16.88	9.21
(default)	Std error	0.52	0.52	0.51	0.51	0.51	0.51
0.6:1.00	Mean	17.27	17.29	16.9	17.65	16.78	8.58
	Std error	0.51	0.52	0.51	0.51	0.52	0.51
0.8:1.00	Mean	18.1	18.01	17.6	18.75	17.84	8.41
	Std error	0.52	0.52	0.52	0.52	0.52	0.52
1.0:1.00	Mean	17.93	17.72	17.71	18.98	17.61	8.52
(normal)	Std error	0.52	0.52	0.52	0.52	0.52	0.52

Table A7
Mean distance errors for changes in the transmural septal conduction velocity ratio.

	Slow transmural septal ratio								
	0.1	0.11	0.12	0.15	0.2	0.25	0.5	0.75	0.9	Norm
Mean	22.19	20.72	18.39	15.94	14.63	14.81	15.62	16.04	16.11	16.18
Std error	0.58	0.58	0.58	0.58	0.58	0.58	0.58	0.58	0.58	0.58

Table A8
Mean distance errors for changes in the septal conduction velocity ratio.

	Slow all septal ratio								
	0.1	0.2	0.25	0.3	0.4	0.5	0.75	0.9	Norm
Mean	45.32	40.3	37.06	34.35	27.44	18.66	15.19	16.5	16.18
Std error	0.78	0.78	0.78	0.78	0.78	0.79	0.79	0.79	0.79

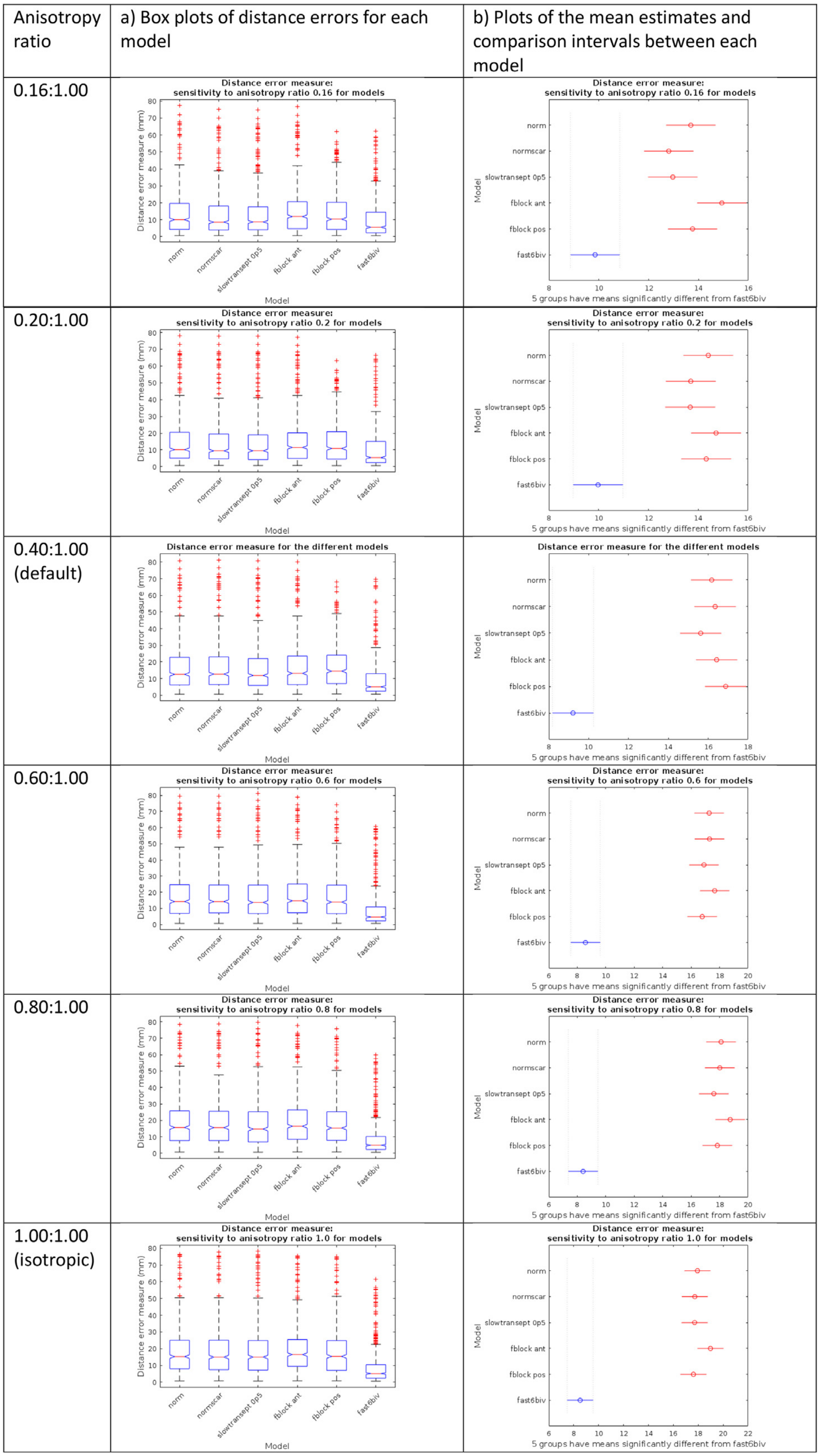

Fig. A14. Plots showing the sensitivity to changes in the anisotropy ratio for the conduction velocity transverse to the fibres: along the fibre directions ranging from 0.16:1.00 to 1.00:1.00 for the different models (Normal: norm, inclusive of scar: normscar, inclusive of slow septal conductivity: slowtransept0p5, inclusive of anterior: fblock ant or posterior functional block: fblock pos, inclusive of fast endocardial conduction: fast6biv).

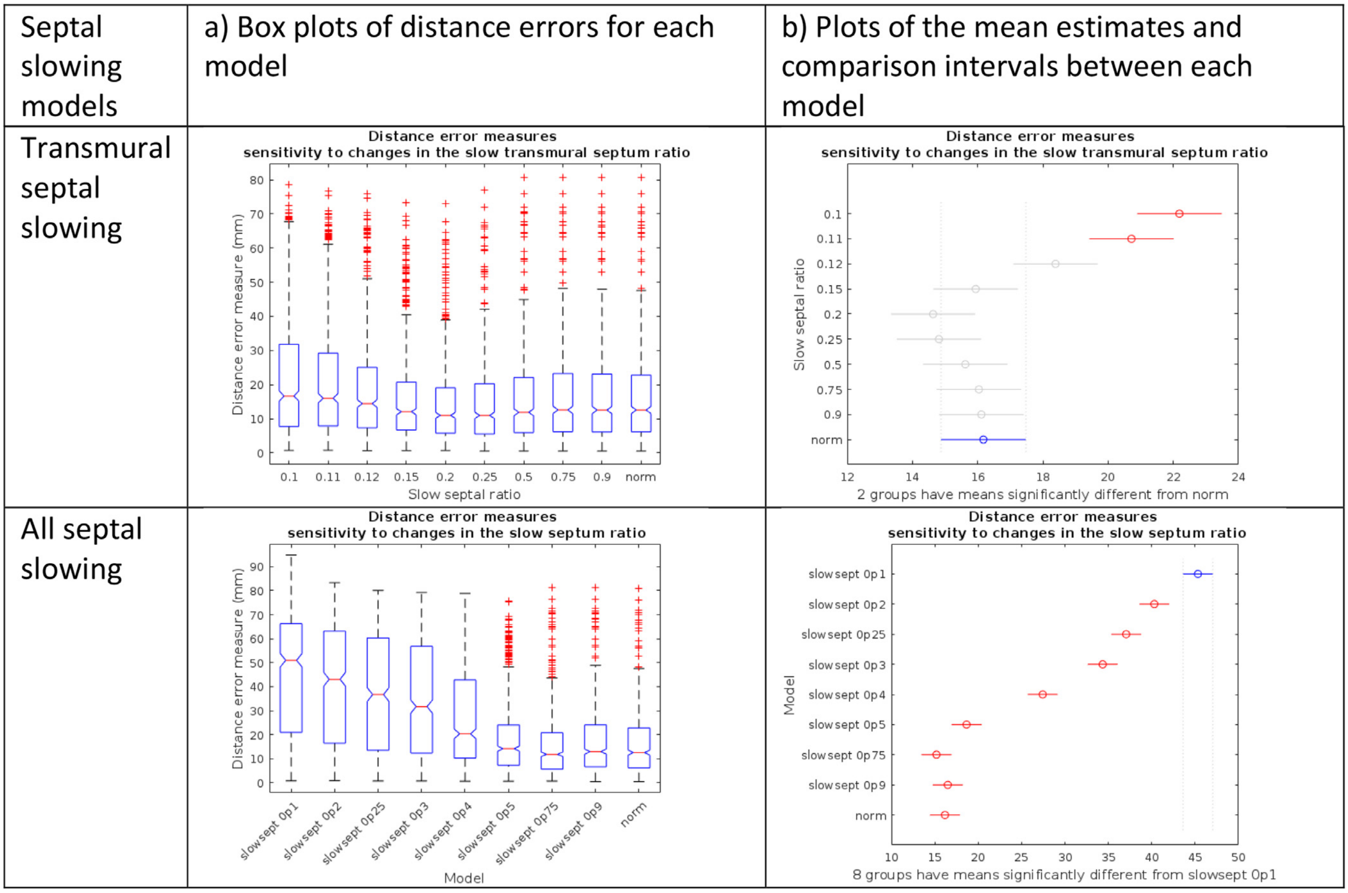

Fig. A15. Plots showing the sensitivity to changes in the slow septal conductivity ratio for transmural slow septal conduction and for slow septal conduction ranging 0.1 to 1.0 (normal).

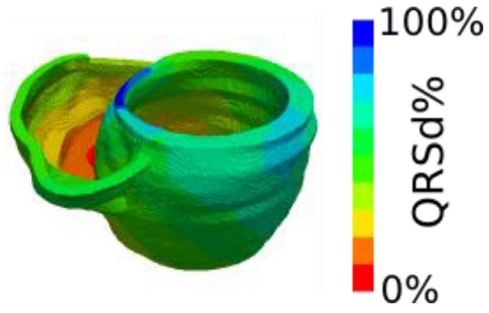

Fig. A16. The latest site for electrical activation is in the septum for models with slow septal conduction where the septal:bulk myocardium conduction velocity ratio falls below 0.15 (where the septal conduction is slowed in the transmural direction) or 0.5 (where the septal conduction is slowed both along and across the myofibres directions).

LBBB, where the latest site of electrical activation occurs on the LV free wall rather than the septum (5), we should not consider models where the transmural septal ratio < 0.15 or septal ratio < 0.5.

Appendix C. Leave one out cross validation

A leave-one-out cross validation approach was used to validate our prediction on the 14 CMR cases that fast endocardial conduction is the best model to use to predict the electrical activation of the heart during RV pacing on the LV epicardial wall. One-way ANOVA found that there were significant differences in the mean temporal and distance errors for the six electrophysiology models for the 13 training cases and for each test case. Tukey post-hoc tests indicated that while posterior functional block and 6-fold FEC had significantly different mean temporal in comparison to the other models, the absolute values of the mean temporal errors were comparable. Tukey post-hoc tests indicated that only fast endocardial conduction had a significantly reduced mean distance error from the rest of the models. The averaged results are presented in Tables A9 and 10.

1. Bayer JD, Blake RC, Plank G, Trayanova NA. A novel rule-based algorithm for assigning myocardial fiber orientation to computational heart models. Ann Biomed Eng. 2012;40(10):2243-54.

2. Streeter DD, Hanna WT. Engineering mechanics for successive states in canine left ventricular myocardium. II. Fiber angle and sarcomere length. Circ Res. 1973;33.

3. Sermesant M, Chabiniok R, Chinchapatnam P, Mansi T, Billet F, Moireau P, et al. Patient-specific electromechanical models of the heart for the prediction of pacing acute effects in CRT: A preliminary clinical validation. Medical Image Analysis. 2012;16(1):201-15.

4. Roth BJ. Electrical conductivity values used with the bidomain model of cardiac tissue. IEEE transactions on bio-medical engineering. 1997;44(4):326-8.

5. Auricchio A, Fantoni C, Regoli F, Carbucicchio C, Goette A, Geller C, et al. Characterization of Left Ventricular Activation in

Table A9
Averaged mean temporal errors for leave-one out cross validation results for the six electrophysiology models (normal: norm, inclusive of scar: scar, inclusive of slow septal conductivity: slowsept, inclusive of anterior: fblock ant or posterior functional block: fblock pos, inclusive of fast endocardial conduction: FEC6).

	Model	Norm	Scar	Slowsept	fblock ant	fblock pos	FEC6
Training	Mean	7.51	7.29	7.92	6.43	−5.06	−7.04
	Std error	0.54	0.54	0.54	0.54	0.54	0.54
Test	Mean	7.6	7.38	8.04	6.58	−5.09	−6.99
	Std error	1.19	1.21	1.19	1.19	1.19	1.19

Table A10
Averaged mean distance errors for leave-one out cross validation results for the six electrophysiology models (normal: norm, inclusive of scar: scar, inclusive of slow septal conductivity: slowsept, inclusive of anterior: fblock ant or posterior functional block: fblock pos, inclusive of fast endocardial conduction: FEC6).

	Model	Norm	Scar	Slowsept	fblock ant	fblock pos	FEC6
Training	Mean	16.19	16.37	15.62	16.41	16.87	9.29
	Std error	0.23	0.23	0.23	0.23	0.23	0.23
Test	Mean	16.18	16.35	15.62	16.42	16.88	9.21
	Std error	0.52	0.52	0.52	0.52	0.52	0.52

Patients with Heart Failure and Left Bundle-Branch Block. Circulation. 2004;109(9):1133-9.
